# Kite-Shaped Molecules Block SARS-CoV-2 Cell Entry at a Post-Attachment Step

**DOI:** 10.3390/v13112306

**Published:** 2021-11-19

**Authors:** Shiu-Wan Chan, Talha Shafi, Robert C. Ford

**Affiliations:** Faculty of Biology, Medicine and Health, School of Biological Sciences, The University of Manchester, Michael Smith Building, Oxford Road, Manchester M13 9PT, UK; talha.shafi@manchester.ac.uk (T.S.); robert.ford@manchester.ac.uk (R.C.F.)

**Keywords:** COVID-19, SARS-CoV-2, anti-viral screening, pseudovirus, spike protein, virus entry, virus attachment, virus post-attachment, pharmacophore

## Abstract

Anti-viral small molecules are currently lacking for treating coronavirus infection. The long development timescales for such drugs are a major problem, but could be shortened by repurposing existing drugs. We therefore screened a small library of FDA-approved compounds for potential severe acute respiratory syndrome coronavirus-2 (SARS-CoV-2) antivirals using a pseudovirus system that allows a sensitive read-out of infectivity. A group of structurally-related compounds, showing moderate inhibitory activity with IC_50_ values in the 2–5 μM range, were identified. Further studies demonstrated that these “kite-shaped” molecules were surprisingly specific for SARS-CoV-1 and SARS-CoV-2 and that they acted early in the entry steps of the viral infectious cycle, but did not affect virus attachment to the cells. Moreover, the compounds were able to prevent infection in both kidney- and lung-derived human cell lines. The structural homology of the hits allowed the production of a well-defined pharmacophore that was found to be highly accurate in predicting the anti-viral activity of the compounds in the screen. We discuss the prospects of repurposing these existing drugs for treating current and future coronavirus outbreaks.

## 1. Importance

COVID-19 has caused an unprecedented public health crisis in recent history. Antivirals are as important as vaccines in fighting SARS-CoV-2 and controlling the pandemic. We need a first line anti-viral defense to reduce the impact of new coronavirus variants while a vaccine is developed, which could improve therapeutics and might be employed as prophylactics in those who cannot be vaccinated or do not respond well to vaccine. This drug, or class of drugs, would target conserved steps in the viral life cycle and be broad-spectrum and generally applicable to COVID variants of the current and next pandemics. By screening an FDA-approved library, we discovered a group of structurally related drugs that inhibit a conserved target, which could be deployed immediately in clinical trials. We have further identified key features of the drugs which can be modeled to re-design the drugs for improved efficacy.

## 2. Introduction

The emergence of the coronavirus disease-2019 (COVID-19) has been a major global challenge that has led to unprecedented efforts to try to control the virus [1]. These measures range from political and societal changes aimed at limiting virus spread, to attempts at eradication, as exemplified by vaccination. Whilst the former measures are highly unpopular, have serious impacts on economic factors, and are of limited effectiveness, vaccination has so far proven to be highly efficient. Nevertheless, vaccine development and vaccination of global populations are lengthy processes. Hence it seems appropriate to look for further control measures for the virus [2]. Missing from the arsenal of effective measures to date has been an effective anti-viral therapy that could be administered prior to, or after acquiring the virus. So far, drug therapy has been limited to attempts to reduce the most life-threatening symptoms of the infection that arise due to over-stimulation of the immune response [3]. We therefore lack a first line anti-viral defense to add to the current toolkit [4]. Such first-line treatments would allow more time to develop new vaccines, could improve therapeutics, and might be employed as prophylactics in those who cannot be vaccinated or do not respond well to vaccine. Such drugs would ideally target conserved steps in the viral life cycle, be broad-spectrum, and therefore generally applicable to COVID variants of the present and future [4].

The post-attachment entry step is one such conserved step [5]. In order to enter host cells to initiate an infection, the virus must recognize and bind to a host cell receptor which then triggers virus–host cell membrane fusion to release the viral nucleocapsid into the host cell cytoplasm [6]. The coronavirus spike protein is divided into an S1 attachment subunit and an S2 fusion subunit [7,8]. The S1 subunits of the severe acute respiratory syndrome virus (SARS-CoV-1) and SARS-CoV-2 share 75% and 50% identity in the receptor binding domain (RBD) and the receptor binding motif, whereas the more conserved S2 subunits share 88% and 100% identity in the fusion domain and fusion peptide [9]. Receptor recognition is not conserved in coronaviruses. They use a range of host receptors. SARS-CoV-2 and SARS-CoV-1 recognize the same receptor, the human angiotensin-converting enzyme 2 (ACE2), whereas the Middle East respiratory syndrome coronavirus (MERS-CoV) recognizes dipeptidyl peptidase 4 (DPP4) [7,8,10,11]. The fusion mechanism, on the other hand, involving the formation of a 6-helix bundle, is conserved amongst viruses [5]. Cleavage at S1/S2 and an internal S2′ site is a pre-requisite to prime fusion in coronaviruses [7,8,12]. SARS-CoV-2 is unusual in that the S1/S2 boundary harbors a furin cleavage site, so the spike protein is already cleaved in the mature virion [7]. Viruses either fuse directly at the host plasma membrane under physiological pH or fuse at the endosome under acidic pH [5]. Members of the coronavirus family can use either or both pathways [13]. There is evidence to suggest that SARS-CoV-2 uses plasma membrane fusion as the default pathway but can use endosomal fusion if the plasma membrane protease, TMPRSS2, is not available; hence, the micro-environment is important in dictating the entry pathway [14]. However, it has been found that infection of ACE2-deficient lung cells depends on clathrin-mediated endocytosis and endosomal cathepsin L, indicating that endosomal fusion may well be the major entry pathway in a subset of cell types [15]. Endosomal fusion is preceded by receptor-mediated endocytosis and trafficking to an acidic compartment to trigger fusion [5]. Clathrin-, caveolae-, and lipid-raft-mediated endocytosis have all been implicated in coronavirus infections [16]. In SARS-CoV-2, both clathrin- and lipid-raft-mediated endocytosis have been demonstrated in two different 293T-ACE2 cell lines, despite somewhat contradictory results [17,18]. There is evidence that SARS-CoV-2 requires phosphatidylinositol 3-phosphate 5-kinase to traffic beyond the early endosome to reach the late endosome/lysosome for cathepsin L-catalyzed S2′ cleavage to trigger endosomal fusion [12,18,19]. Thus, the post-attachment entry steps depend heavily on a number of host signaling molecules which are amenable for drug targeting. Targeting conserved viral and/or host factors/processes negates the problematic drug escape mutants and is a current trend of generating broad-spectrum anti-virals [20].

Our aim was to find drug hits that target the entry steps, in particular the post-attachment step but any attachment blockers can be useful in virus-specific inhibition or universal synergistic inhibition with post-attachment inhibitors. Hence, we employed a pseudovirus system in which the mouse leukemia virus (MLV) is pseudotyped with the SARS-CoV-2 spike protein, which would allow us to specifically screen for entry inhibitors [21,22]. During the current COVID crisis our first aim was to explore re-purposing of FDA-approved drugs and natural products, with the goal of using any hits to generate a pharmacophore to inform next generation drug design.

## 3. Experimental Procedures

### 3.1. Cells

293T, 293T-ACE2, A549-ACE2, Caco2, Huh-7, and Vero cells were cultured in Dulbecco’s modified Eagle’s medium with 4 mM of glutamate (DMEM; SigmaAldrich, Gillingham, UK) and supplemented with 10% fetal calf serum (FCS; Sigma), 100 units/mL of penicillin, and 100 μg/mL of streptomycin (Sigma) at 37 °C, 5% CO_2_. The culture medium of A549-ACE2 was supplemented with 1 μg/mL of puromycin (Sigma). The culture medium of Caco2 and Huh-7 was supplemented with 1× non-essential amino acid. Calu3 cells were cultured in alpha-minimal essential medium supplemented with 2 mM of glutamate.

### 3.2. Pseudovirus System

293T cells seeded at 4 × 10^6^ per 100-mm dish were co-transfected with 6 μg of a plasmid encoding MLV gag-pol, 8 μg of the transfer vector encoding a luciferase reporter, and 6 μg of a plasmid encoding an empty vector, a viral envelope glycoprotein SARS-CoV-1-S, SARS-CoV-2-S, MERS-CoV-S, or VSV-G using calcium phosphate (125 mM of CaCl_2_, 0.7 mM of Na_2_HPO_4_, 70 mM of sodium chloride, 25 mM of Hepes, pH 7.05) (see Supplementary Information Figure S1). The medium was replaced with fresh medium after 24 h and supernatant containing pseudoviruses was harvested after 48 h, clarified by centrifugation at 1000 rpm/4 °C for 10 min, filtered through a 0.45-μm filter, and stored at −80 °C. The resulting pseudovirus contains an MLV gag-pol backbone packaging a luciferase reporter genome and displaying one of the viral envelope glycoproteins. The MLV pseudotyped with an empty vector was bald.

### 3.3. Anti-Viral Drug Screening

Two libraries from APExBIO containing 1363 FDA-approved drugs (cat: L1021) and 137 natural compounds (cat: L1039) were used in screening. Drug stocks at 10 mM were diluted into 1 mM in their own solvents or diluted directly into medium to 20 μM. 293T-ACE2 cells seeded at 25,000 cells per well of 96-well plates were pre-treated with 10 μM of individual drugs, in duplicate, for 1 h. After 1 h, 25 μL of pseudovirus was added together with drugs to maintain the final drug concentration at 10 μM. Positive controls of spike pseudovirus treated with solvents (DMSO, ethanol, or water) and a negative control of bald pseudovirus (without spike) were included in each screen. A no cell control treated with DMSO was used as a background control. A parallel set of 96-well plates was set up with only drugs without pseudovirus, in duplicate, to test for drug cytotoxicity. After incubation for 37 °C, 5% CO_2_ for 48 h, they were tested for luciferase activity for % infectivity relative to the infected, solvent controls and for % viability relative to the un-infected solvent controls. For the generation of concentration curves, serial dilutions of drugs were titered, in duplicates and IC_50_ was calculated using Prism9 (GraphPad, San Diego, CA, USA).

### 3.4. Luciferase Assay

Cells were lysed by the addition of 100 μL of passive lysis buffer (Promega) to each well and shaken for >15 min. Luciferase assay was carried out as described in [23,24,25] in a buffer containing 0.0165 M of glycylglycine, 0.01 M of MgSO_4_, 2.65 mM of EGTA, 10.5 mM of potassium phosphate, 1.4 mM of adenosine 5′-triphosphate, 0.86 mM of dithiothreitol (DTT), 0.175 mg/mL of bovine serum albumin, and 0.035 mM of luciferin (Promega) using 50 μL of the lysate and a luminometer (Berthold Technologies GmbH & Co. KG, Bad Wildbad, Germany).

### 3.5. XTT Viability Assay

Cell viability was measured by addition of 50 μL of 1 mg/mL 2,3-Bis-(2-Methoxy-4-nitro-5-sulfophenyl)-2H-tetrazolium-5-carboxanilide, disodium salt (XTT, Biotium, Fremont, CA, USA) and 20 μM of N-methyl dibenzopyrazine methyl sulfate (Cayman) in culture medium to each well for 2–4 h at 37 °C/5% CO_2_. Absorbance was read at 450 nm with a reference wavelength of 650 nm using a plate reader (Bio-Tek Synergy HT, Winooski, VT, USA).

### 3.6. Time-of-Addition Experiment

In time-of-addition experiment, drugs were added at different times of infection. First, 25 μL of pseudovirus was added to each well for 1 h, then washed off twice with PBS. Inhibition of the entry steps was assayed by the addition of drugs during the 1 h infection with and without an 1 h pre-treatment. Drugs were washed off together with the virus at 1 hpi and incubation continued until 48 hpi in the absence of drugs. Inhibition of post-entry steps involved no drug pre-treatment and infection in the absence of drug. After washing off the virus at 1 hpi, drugs were added immediately or at 2 hpi and were present for the duration of the rest of the 48-h infection. A full-time treatment was included as a control in which drugs were added 1 h pre-infection and during infection. After washing off the drugs and viruses at 1 hpi, fresh drugs were added until 48 hpi.

### 3.7. Temperature Shift Experiment

Cells seeded on 96-well plates were pre-cooled on ice for 1 h. For the attachment assay, drugs were diluted into pre-cooled viruses on ice to 10 μM just before infection and the 100 μL virus–drug mix was added to each well for 1 h. After 1 h, the wells were washed 3× with ice-cold medium. Warm medium was added, and incubation continued at 37 °C/5% CO_2_ until 48 hpi. For the penetration (post-attachment) assay, 100 μL of pre-cooled virus without drugs were added to each well for 1 h infection. After 1 h, the wells were washed 3× with ice-cold medium. Drugs diluted in warm medium were added and incubated at 37 °C, 5% CO_2_ for 1 h. After 1 h, the drugs were washed off with 1× warm PBS (without Mg^2+^, Ca^2+^) and 1× with warm medium. Fresh, warm medium was added, and incubation continue at 37 °C, 5% CO_2_ until 48 hpi.

### 3.8. Western Blotting

Western blotting was performed as described [23,24,26,27,28]. Protein lysates were harvested into a radioimmunoprecipitation assay (RIPA) buffer (50 mM of Tris Ph 8.0, 150 mM of NaCl, 1% NP40, 0.5% Na deoxycholate, 0.1% SDS) plus protease (Sigma) and phosphatase inhibitors (APExBIO, Houston, TX, USA). Proteins from equal number of cells were separated on TGX Stain-Free SDS-PAGE gel (Bio-Rad, Hercules, CA, USA), transferred to polyvinylidene difluoride membranes (Millipore, Burlington, MA, USA), blocked in 5% semi-skimmed milk (Marvel) in 0.1% Tween 20 (Sigma)/TBS (50 mM of Tris pH 7.4, 150 of mM NaCl) before being probed against primary and horseradish peroxidase (HRP)-conjugated secondary antibodies in blocking buffer. Anti-ACE2 antibody (Proteintech, Rosemont, IL, USA) was used at 1:2000 and anti-mouse HRP (Cell Signaling Technology, Danvers, MA, USA) at 1:1000. Anti-SARS-CoV-2 spike antibody (BEI Resources NR-52947) was used at 1:1000 and anti-rabbit HRP (Cell Signaling Technology) at 1:1000. Protein bands were detected using Clarity^TM^ ECL substrate (Bio-Rad). Images were captured and quantified using ChemiDoc^TM^ XRS+ system (Bio-Rad) and ImageLab 6.0.1 software (Bio-Rad).

### 3.9. Pharmacophore and Docking Studies

A pharmacophore was built using the pharmacophore query module implemented in Molecular Operating Environment (MOE) [29]. In brief, compounds within 0.5 log fold activity of the highest inhibitory activity were considered active, whereas compounds yielding infectivity ranging above 100% infectivity (control) values were considered inactive. These compounds were stochastically searched for conformers using default parameters and packed as a known dataset during pharmacophore development. Docked conformations of asenapine due to its fused ring rigid structure were considered as templates for pharmacophore development. The pharmacophoric features were calculated using AutoPH4 script [30] with holo conditions, and manually optimized for maximum performance. The final model was used on the complete drug dataset to access the screening performance.

Docking studies were performed using GOLD software version 2020.3.0 [31]. In summary, 2D depicted structures of compounds were extracted from the PubChem database [32] and were compiled in an MOE database. The dataset was washed for adjuvant atoms and protonated at pH 7.4. Partial charges were computed using Merck molecular force field 94x (MMFF94x) methods as implemented in MOE [29]. The charge calculated ligands were energy minimized for relaxed three-dimensional conformations. The LeuBAT structure with clomipramine (4MMD) [33] and the SLC6a19 structure (6M18) [34] were downloaded from the PDB database. The structural discrepancies in the models were corrected using the structure correction module implemented in MOE. The corrected structures were protonated, energy minimized, and saved for docking simulations. Both protein and ligands were considered flexible during the simulations. To explore diversity of conformational solutions, a 15 Å area around the ligand binding site was selected. Furthermore, the cavity was strictly restricted to solvent accessible area using LIGSITE implemented in GOLD. To ensure reproducibility, a total of 100 conformations were generated and ranked according to the scoring function.

### 3.10. Statistical Analysis

Statistical analysis was performed, and graphs were plotted using Prism 9.0 (GraphPad). A Shapiro–Wilk normality test and one sample *t*-test were used for the analysis of temperature shift, infectivity, and viability data against a theoretical mean of 100. Two-way ANOVA and Tukey’s post hoc test were used for the analysis of time-of-addition data and comparison of various pseudoviruses. A *p*-value of <0.05 was considered statistically significant.

## 4. Results

### 4.1. 293T-ACE2 Is a Suitable Cell Type for Pseudovirus Drug Screening

In order to re-purpose drugs for fast-tracking COVID-19 prophylaxis and treatments, we undertook screening of two libraries of FDA-approved drugs and natural products from APExBIO by using MLV pseudotyped with the SARS-CoV-2 S protein, with the goal of targeting the major attachment and entry steps (Supplementary Information Figure S1). The spike protein is derived from the Wuhan-Hu-1 SARS-CoV-2 and has been codon optimized for mammalian expression [7]. To find a suitable human cell type for the screening of drugs inhibiting viral infectivity we tested SARS-CoV-2-S, SARS-CoV-1-S, MERS-CoV-S, and vesicular stomatitis virus (VSV)-glycoprotein (G) pseudoviruses against a range of cell types and employing the pseudovirus-encoded luciferase activity as a read-out for infectivity (Supplementary Information Figure S2). As expected, the control VSV-G pseudovirus, which has a broad host range [35], infected all cell types. SARS-CoV-1-S and SARS-CoV-2-S pseudoviruses did not infect the hepatocyte cell line, Huh-7. The heterogeneity in ACE2 expression in Huh-7 cell populations together with the widely varied characteristics of different laboratory-passaged Huh-7 lines may explain the discrepancy in the susceptibility of Huh-7 cells to native SARS-CoV-2 infection [8,36,37,38]. In contrast, MERS-CoV-S pseudovirus, which preferentially binds DPP4 as a receptor rather than ACE2 [11], showed a high level of infectivity in Huh-7 cells. The green African monkey Vero cells (kidney), human colorectal epithelial Caco2 cells, and human lung epithelial Calu3 cells all express a high level of ACE2 and are susceptible to native SARS-CoV-2 infection [37] but only Vero cells could be infected to a high degree by SARS-CoV-1-S and SARS-CoV-2-S pseudoviruses in this study. There was also a high background luciferase read-out from the empty (bald) pseudovirus in Calu3 cells. The human kidney epithelial 293T cells and human lung epithelial A549 cells express a low level of native ACE2 [37]. A549 cells stably expressing recombinant human ACE2 showed SARS-CoV-1-S and SARS-CoV-2-S pseudovirus infectivity, but not to the same high level as 293T-ACE2 cells, which were therefore employed for initial drug screening. Compared to 293T cells not overexpressing ACE2, infectivity of SARS-CoV-2-S pseudovirus in 293T-ACE2 cells was about 100× higher (Supplementary Information Figure S3a,d). Quantitation by Western blot estimated the number of ACE2 receptors to be at least ten times higher in 293T-ACE2 cells than in the untransfected 293T cells with no loss of ACE2 expression in late passaged cells (P16) compared to early passaged cells (P4) (Supplementary Information Figure S3b,d). The number of trimeric spike proteins present on the surface of the pseudovirus in each infection experiment was also estimated (Supplementary Information Figure S3c,d). The data imply that the 293T-ACE2 cells’ ACE2 receptors will outnumber spike protein in the pseudovirus infection experiments (Supplementary Information Figure S3b–d), which is likely to be representative of the in vivo situation, especially at early stages of infection. Furthermore, the quantitation confirmed that the concentrations of proteins in the assays described below were well below the drug concentrations used. This was important to allow for the possibility of full inhibition by any given drug that was working by blocking the ACE2-Spike interaction and hence virus attachment to the cell.

### 4.2. Screening of Two Libraries Identifies Drug Hits

**Figure 1:**
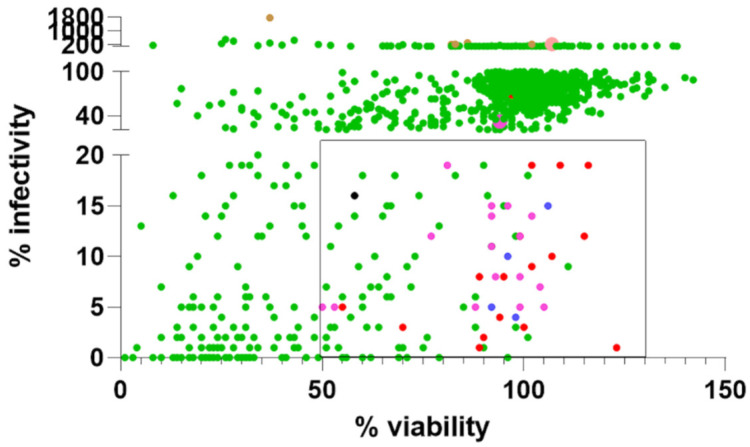
Kite-shaped molecules predominates drug hits. A scatter plot of % infectivity versus % viability in two library screens against SARS-CoV-2 spike protein pseudovirus infection of 293T-ACE2 cells. Individual data are represented by green dots. Ninety-eight drugs within the square (≤20% infectivity; ≥50% viability) were negative screened against VSV-G pseudovirus apart from the one represented by a black dot. Drug hits specific for SARS-CoV-2 spike pseudovirus are represented by red dots, with kite-shaped molecules represented by violet dots and kite-like-shaped molecules represented by blue dots. Imipramine fell outside the range of the square in the library screen (represented by an enlarged violet dot) but displayed a higher inhibitory effect in the focused kite-shaped molecules screen to make the top hits (see Table S3). Some drug hits displayed varied viability. Select drugs showing ≥200% of SARS-CoV-2 pseudovirus infectivity were also negative screened against VSV-G pseudovirus (represented by brown dots) and tazarotene was found to be a true potentiator specific for SARS-CoV-2 spike pseudovirus and is represented by an enlarged pink dot. Some dots overlap.

To rapidly re-purpose drugs as anti-virals, an initial screen of two libraries of 1363 FDA-approved drugs and 137 natural products was carried out using the above-mentioned cell line. The compounds at 10 μM were incubated with the cells after dilution of the drugs into cell growth media from (predominantly) DMSO-solubilized stock solutions or (occasionally) ethanol-based or water-based stocks. Any cytotoxicity effects of the drugs at this concentration were controlled for using an XTT cell viability assay in un-infected cells. The results are summarized in Figure 1. One hundred and one potential drug hits with ≤20% infectivity and ≥50% viability were identified. Because the pseudovirus is a hybrid between MLV and SARS-CoV-2, the drug hits could potentially be false positives by inhibiting the post-entry steps of transcription and translation mediated by the MLV. Therefore, we performed a negative screen against MLV pseudotyped with the VSV-G, which shares common MLV post-entry steps but differs in receptor recognition and entry mechanisms [13,39]. Compounds that also inhibit VSV-G pseudovirus are most probably inhibiting MLV transcription and translation although it remains possible that some of them may target an entry step common to SARS-CoV-2 and VSV as can be seen from the range of VSV-G pseudovirus infectivity and the differential inhibition of the two pseudoviruses. Compounds scoring <65% infectivity in a negative screen against VSV-G pseudotyped virus are arbitrarily considered false positives. As expected, reverse transcriptase inhibitors (e.g., tenofovir, zidovudine), integrase inhibitors, nucleoside analogues, purine and pyrimidine synthesis inhibitors, transcriptional and DNA replication inhibitors potently inhibited VSV-G pseudovirus infectivity indiscriminately and are likely false positives. Other false positives include tubulin and poly-ADP ribose polymerase (PARP) inhibitors.

After excluding false positives, there are 36 top hits (Figure 1, Table 1). Using a pseudovirus, this study simply tested drugs that can inhibit virus entry (i.e., spike binding to the ACE-2 receptor, endocytosis, and/or fusion), so other steps in the replication cycle were not evaluated. The cathepsin inhibitor, E64d, returned as a hit (9% infectivity), whereas the TMPRSS2 inhibitor, camostat mesylate, is not a hit (120% infectivity). This is in agreement with the utilization of the endosomal but not plasma membrane entry pathway in 293T-ACE2 cells [17,18]. Two new hits are identified amongst the most potent: vandetanib (1%; 89% viability) and pimavanserin (3%; 70% viability), which target tyrosine kinase and serotonin 5-hydroxytryptamine (HT) receptor, respectively [40,41]. Interestingly, many tyrosine kinase inhibitors returned as false positives, i.e., they also affect VSV-G pseudotyped virus infectivity. Several inhibitors of serine/threonine/tyrosine kinases and cyclin-dependent kinases (CDKs) with similar structures to vandetanib are false positives, indicating the importance of the exact chemistry in inhibition (Table S1).

The hits are dominated by a group of kite-shaped (17 hits) and kite-like molecules (4 hits) which make up 60% of the top drug hits (Figure 1). The kite-shaped molecules are a class of molecules that displayed a similar structure and a shape reminiscent of a traditional Chinese kite. These had a well-conserved tri-cyclic core structure (forming the sail of the kite) and a more variable extension from the central 6- or 7-membered ring (forming the tail of the kite) (Table 1). They are mostly anti-psychotics, anti-depressants, and antihistamines that target the serotonin 5-HT, dopamine, H1 histamine, muscarinic, and adrenergic receptors. Amongst them chlorprothixene is the most potent kite-shaped molecule (5% infectivity), whereas azelastine is the most potent kite-like-shaped molecule (4% infectivity). Structurally distinct serotonergic and muscarinic antagonists are also found amongst the top hits (pimavanserin) and the hits listed below. Agonists, histamine, acetylcholine, serotonin, did not enhance infectivity.

The rest of the hits are made up of one, or a few members of, estrogen receptor modulators, protein kinase C (PKC)/mitogen-activated protein kinase (MAPK) inhibitors, calcium channel/P-glycoprotein blockers, anti-cancer agents, and anti-fungals. Amongst serine/threonine kinase inhibitors, PKC/MAPK inhibitors returned as hits, but the CDK inhibitors are likely false positives. Interestingly, the CDK inhibitors, palbociclib HCl and palbociclib isethionate, displayed a large discrepancy in inhibiting VSV-G pseudovirus infectivity (63% and 32%, respectively), indicating a part played by the salt. Two estrogen receptor modulators, with similar structures, returned as a hit and a false positive, again indicating the importance of the chemistry in anti-viral (Table S2). Agonists, estradiol, estriol, and antagonist progesterone did not enhance infectivity. The multi-functional anti-viral, Arbidol, which is in use in Russia and China in treating influenza patients and has entered into COVID trials [42], is modestly inhibitory (18% infectivity). It is excluded from the hit list because of a marginally low VSV-G pseudovirus infectivity at 62%.

SARS-CoV entry requires Abelson kinase and is inhibited by imatinib [43]. However, imatinib, as a free base, did not inhibit SARS-CoV-2-spike pseudovirus in our screen (81% infectivity, 103% viability) although this may be a confounding effect of its poor water solubility as imatinib, as a mesylate salt, is soluble in water and toxic (1% infectivity, 22% viability). It has been reported that SARS-CoV-2 entry is facilitated by binding to surface heparin and hence is inhibited by exogenous heparin/heparin sulfate [44]. However, we did not detect significant inhibition of heparin sodium on SARS-CoV-2-spike pseudovirus infectivity (88% infectivity; 98% viability). This indicates that the ectopic expression level of ACE2 is high enough to mediate virus entry in vitro and does not require facilitation by heparin.

Only one of the natural compounds returned as hits (Table 1). Timosaponin A3 is anti-cancer but has a high toxicity (5% infectivity; 55% viability). The most potent, tetrandrine (1% infectivity) and oridonin (9% infectivity), inhibited VSV-G pseudovirus infectivity to 39% and 37% and were therefore considered false positives in our screen although tetrandrine was found to be inhibitory to native SARS-CoV-2 infection in Vero cells in another screen [45]. However, the differential increase in infectivity of VSV-G pseudovirus may mean they target a common entry step in SARS-CoV-2 and VSV. Cepharanthine has been identified as a potent anti-SARS-CoV-2 in several other screens [45,46]. With an infectivity of 1% for both SARS-CoV-2-spike and VSV-G pseudoviruses, cepharanthine is a false positive in our screen. Our results indicate that the anti-viral effect of cepharanthine is not at the entry step.

We also identified six compounds that potentiated SARS-CoV-2-spike infectivity by more than 200% for negative screens and those with scores >100% in VSV-G pseudovirus assays considered false positives. Most of these six potentiators turned out to be non-specific enhancers of transcription e.g., the histone deacetylase (HDAC) inhibitors increased transcription of the reporter indiscriminately. Only tazarotene, which did not show any potentiating effect on VSV-G pseudovirus infectivity, was considered a true potentiator. Tazarotene is a synthetic topical retinoid which induces expression of the tumor suppressor, tazarotene-induced gene 3 [47]. Drospirenone may be a true potentiator because of its differential (1774%) increase of SARS-CoV-2-spike pseudovirus infectivity compared to (381%) of VSV-G pseudovirus infectivity. Drospirenone binds to the progesterone receptor and the activated complex then binds to specific sites on DNA [48]. The possibility that drospirenone is a true potentiator is strengthened by the presence of estrogen/progesterone modulators amongst the inhibitory drug hits which together may indicate a role of regulating estrogen/progesterone receptors in SARS-CoV-2 entry.

### 4.3. Kite-Shaped Molecules Inhibit SARS-CoV-2 Pseudovirus Infection

Because of a prevalence of inhibitory activity found within the kite-shaped molecules, we therefore selected all of 61 kite-shaped molecules from the two libraries and tested them at the same time at 10 μM so that we could rank them in order of inhibitory activity. Five that were cytotoxic were excluded at this stage; the remaining molecules showed a range of activity against pseudovirus infectivity. We ranked them in order of inhibition of infectivity, from which we constructed a pharmacophore and picked our top hits for further analysis. Supplementary Table S3 summarizes the experimental data for the kite-shaped molecules.

**Figure 2:**
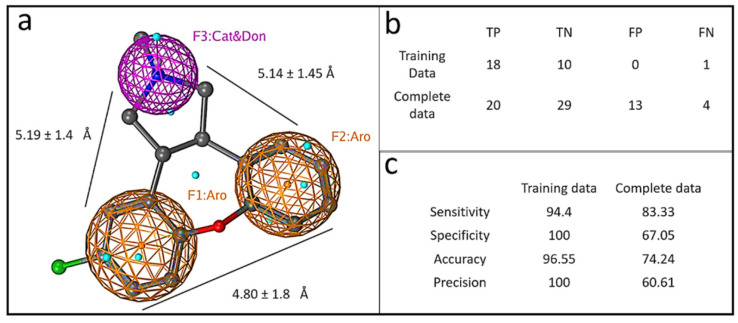
Graphical depiction of pharmacophore model. (**a**) A three point pharmacophore model based on the kite-shaped molecules. The asenapine structure is superimposed (ball and stick representation) for comparison. Brown mesh represents aromatic moieties (Aro) and magenta mesh represents a H-bond donor/cation group (Cat&Don). Small spheres (cyan) highlight features in asenapine that are not relevant for the overall pharmacophore. (**b**) Displays the numbers of true (T) and false (F) positive (P) and negative (N) hits within the datasets that are discriminated by the pharmacophore (see Methods). Panel (**c**) summarizes the pharmacophore model performance.

The similarity in the overall structure of the kite-shaped molecules allowed the generation of a pharmacophore (Figure 2). We chose the second top hit, asenapine, because the top hit chlorprothixene was slightly cytotoxic. Asenapine is a kite with a shorter tail. Pharmacophores for tricyclic antidepressants (TCAs) have previously been described, showing the importance of the two outer aromatic rings, one of which is more hydrophobic. The tail region in the pharmacophore shows H-bonding propensity and the ability to form a positive charge on an amine group [49,50]. The pharmacophore generated from the SARS-CoV-2 infectivity assay displayed similar features to these prior studies (Figure 2a) but with more tightly defined distances and angles between the three main pharmacophore features. The three-feature minimal model was able to classify active compounds with predictivity values above 90%. When the complete continuous data was split into active and non-active compounds based on log activity values, the model still showed F score values of 70%. The model showed excellent predictive ability with both training and complete datasets (Figure 2b,c). The pharmacophore predicted 12 out of 31 top hits (excluding chloroquine and derivatives, azithromycin and derivatives, and E64d) when protein structures were included in the pharmacophore; and 15 out of 31 top hits when protein structures were excluded. It correctly predicted eight out of 14 top kite hits and top kite-like molecules including azelastine. Interestingly, the pharmacophore predicted three more top hits with targets other than dopamine, histamine, and serotonin receptors. They include kite-like estrogen receptors, raloxifene and tamoxifen, and a kite-like PKC inhibitor, sotrastaurin. Remarkably, it correctly predicted one of our top hits, raloxifene, but not a structurally similar bazedoxifene, which was a non-hit in our assay.

### 4.4. Kite-Shaped Molecules Are Amongst the Most Efficacious of the Top Hits

**Figure 3:**
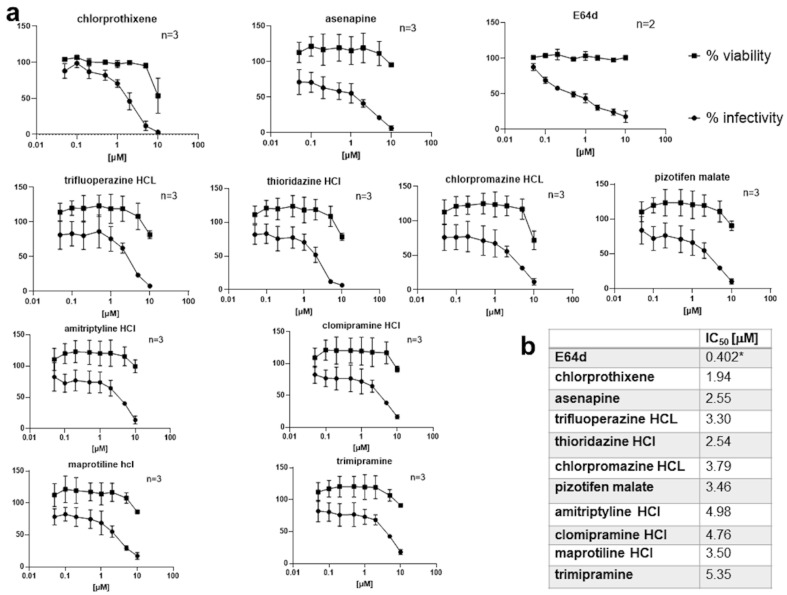
Dose–response curves of kite-shaped molecules in SARS-CoV-2-S inhibition. Mouse leukemia virus pseudotyped with spike protein (S) from severe acute respiratory syndrome coronavirus-2 was used to infect 293T-ACE2 cells in 96-well plates for 48 h in the presence of serial doses of the drug, as indicated, with 1 h pre-treatment. (**a**) Infectivity was measured as luciferase activity and expressed as % infectivity to infected, own solvent control (dimethyl sulfoxide, ethanol or water). Viability was measured by XTT assays in uninfected cells and expressed as % viability to un-infected solvent control (dimethyl sulfoxide, ethanol or water). Data are presented as mean ± SD of two to three repeats, as indicated. The square symbols are for cell viability (% of control) and the round symbols are for infectivity by the pseudovirus (% of control). (**b**) Summary of IC_50_ values. * The IC_50_ of E64d was obtained by interpolation from the dose–response curve.

Although all the prior screening had been done with 10 μM concentrations of compounds, to further explore the efficacy of the kite-shaped molecules, we generated dose-response curves using a range of drug concentrations from 10 to 0.05 μM (Figure 3a and Figure S4). The kite-shaped molecules displayed IC_50_ values from 1.9 to 5.4 μM, compared to 0.4 μM for E64d (Figure 3b). Compared to other known top hits, the kite-shaped molecules had the lowest IC_50_ and were the most efficacious amongst the new hits (Figure 3b and Figure S4). All the kite-shaped drugs showed *de minimis* cytotoxicity at 5 μM and even at the highest drug concentration employed, the cells generally displayed a viability above 76% with few exceptions (Figure 3a). Since we diluted the drugs in cell growth medium, a confounding effect on the determination of IC_50_ could be water solubility of the drugs. Most of the drugs are readily water soluble in their charged state (pizotifen malate is the exception), but they may partition into the cell membrane via their uncharged forms which will have very low water solubility (Supplementary Table S4).

### 4.5. Kite-Shaped Molecules Target Common Coronaviral Entry Step(s)

**Figure 4:**
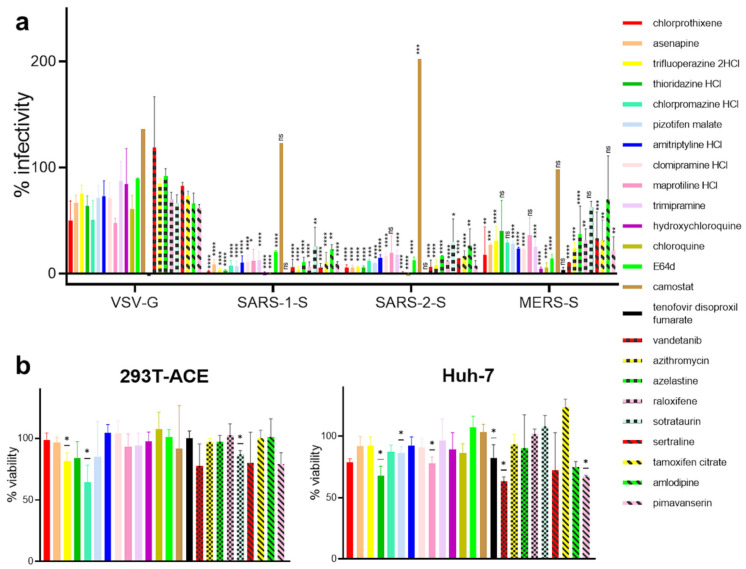
Kite-shaped molecules specifically inhibit coronavirus infection. Mouse leukemia virus pseudotyped with glycoprotein from vesicular stomatitis virus (VSV-G) and spike protein (S) from severe acute respiratory syndrome coronavirus (SARS-1-S), SARS-CoV-2 (SARS-2-S), and Middle East respiratory syndrome coronavirus (MERS-S), was used to infect 293T-ACE2 cells, in a 96-well plate for 48 h in the presence of the drug, as indicated, with 1 h pre-treatment. (**a**) Infectivity was measured as luciferase activity and expressed as % infectivity versus infected, solvent control. Data are presented as mean +/− SD of two-three repeats. Statistically significant differences compared with VSV-G pseudovirus are analysed by 2-way ANOVA and are represented by * *p* < 0.05, ** *p* < 0.01, *** *p* < 0.001, and **** *p* < 0.0001. ns = non-significant. The differences of % infectivity between SARS-CoV-1-S or SARS-CoV-2-S with MERS-CoV-S pseudoviruses are significant in trifluoperazine, thioridazine, sotrastaurin, and amlodipine treatments (not shown). (**b**) Viability was measured by XTT assays in uninfected samples and expressed as % viability versus solvent control. Data are presented as mean +/− SD of two-four repeats. Statistically significant differences are analyzed by one-sample *t*-test and are represented by * *p* < 0.05.

To study whether the kite-shaped molecules and other top hits also target other coronaviruses, we used MLV pseudotyped with the SARS-CoV-1 and MERS-CoV spike protein, respectively, using VSV-G pseudovirus as a negative control. We also included the fourteenth ranked kite-shaped hit, trimipramine, because it had previously been identified as a specific SARS-CoV-1 entry blocker and an inhibitor of native SARS-CoV-2 infection [21]. As expected, the reverse transcriptase inhibitor, tenofovir disoproxil fumarate, completely inhibited infection of all pseudoviruses indiscriminately (Figure 4). The lysosomotropic agents, chloroquine and hydroxychloroquine, inhibit late endosomal/lysosomal fusion but not early endosomal fusion [51]. E64d is a cathepsin inhibitor in the late endosome [52]. As expected, they inhibited SARS-CoV-1, SARS-CoV-2, and MERS-CoV infections facilitated by late endosomal/lysosomal fusion, but not VSV-G infection facilitated by early endosomal fusion [13,39]. Camostat, a plasma membrane TMPRSS2 inhibitor, on the other hand, did not inhibit any of the pseudovirus infections. The majority of the selected kite-shaped molecules and other new hits showed effects similar to those of chloroquine, hydroxychloroquine and E64d by specifically inhibiting infectivity of the SARS-CoV-2-S, SARS-CoV-1-S, MERS-CoV-S, but not VSV-G pseudotyped viruses, suggesting that they target late entry steps specific to these three coronaviruses. Exceptions are trifluoperazine, thioridazine, sotrastaurin, and amlodipine, which displayed significant inhibition of SARS-CoV-1-S and SARS-CoV-2-S infections but non-significant inhibition of MERS-CoV-S infection compared to VSV-G pseudovirus infection. The low levels of inhibition of VSV-G pseudovirus infectivity for a few of the compounds could be due to inhibition of common post-attachment pathways with VSV-G. For example, chlorpromazine is a known inhibitor of clathrin-mediated endocytosis of VSV [39]. The kite-shaped molecules and other top hits inhibited SARS-CoV-1-S and SARS-CoV-2-S pseudoviruses equally well and the inhibition was 1.4 to 15-fold higher than that for MERS-CoV-S pseudovirus, suggesting that they may, in addition, discriminate between different receptor-mediated pathways for viral entry. These results imply that the kite-shaped molecules target coronaviral-specific entry steps, most likely a post-attachment step shared amongst SARS-CoVs and MERS-CoV.

### 4.6. Kite-Shaped Molecules Inhibit Pseudovirus Entry

**Figure 5:**
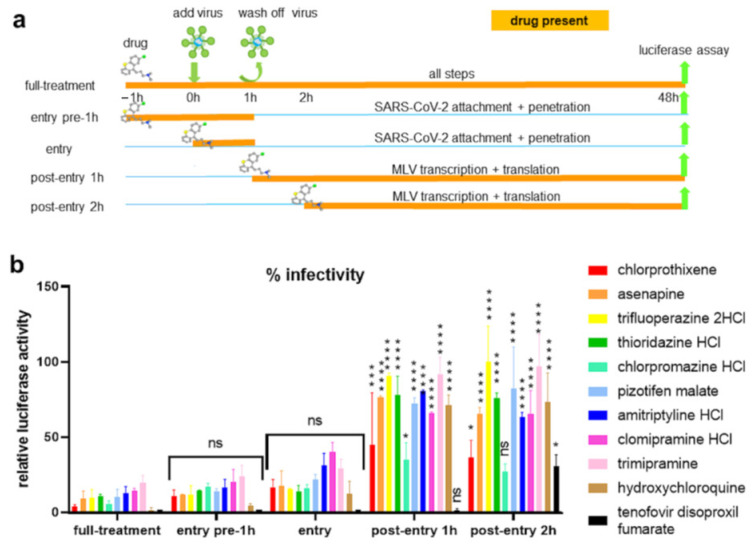
Kite-shaped molecules inhibit SARS-CoV-2 pseudovirus at entry steps. Mouse leukemia virus (MLV) pseudotyped with spike protein (S) from severe acute respiratory syndrome coronavirus-2 (SARS-CoV-2) was used to infect 293T-ACE2 cells in 96-well plates for 48 h in a time-of-addition experiment. (**a**) Schematic of time-of-addition experiment. Full-time treatment involved 1 h drug pre-treatment and 1 h infection in the presence of drug followed by drug and virus wash-off and addition of fresh drug for the rest of 48 h. Entry assay involved 1 h infection in the presence of drug with and without 1 h drug pre-treatment. The drug and virus were washed off and fresh medium was added without drug for the rest of 48 h. Post-entry assay involved no drug pre-treatment and infection in the absence of drug. Following virus wash-off, drug was added at 1 h post-infection (hpi) or 2 hpi for the rest of 48 h. The thick yellow bar indicates the stages when drug is present. Addition of drug is represented by a drug image obtained from PubChem. (**b**) Infectivity was measured as luciferase activity and expressed as % infectivity to infected, own solvent control (dimethyl sulfoxide, ethanol, or water) at the same time point. Data are presented as mean +/− SD of two repeats. Statistically significant differences compared with full-time treatment are represented by * *p* < 0.05, *** *p* < 0.001 and **** *p* < 0.0001. ns = non-significant.

To study the mechanisms of inhibition in greater detail, we undertook a time-of-addition experiment in which drugs were added at different time points during infection (Figure 5a) with the hypothesis that the time point(s) may distinguish different step(s) that may be inhibited by the drugs. As before, we studied the top nine hits together with trimipramine. The kite-shaped molecules were able to inhibit infection when added during the entry step (with and without 1 h pre-incubation), to similar extents to the full-treatment (Figure 5b). Most of the kite-shaped molecules did not inhibit infectivity when only added 1 and 2 h post-infection (hpi). This observation was similar for the entry blocker, hydroxychloroquine, suggesting that the kite-shaped molecules are also entry blockers. The exceptions were chlorprothixene and chlorpromazine, which were still able to reduce infectivity to 45% and 35%, respectively, when added at 1 hpi. However, these reductions in infectivity were still much lower than the complete inhibition of infectivity observed for the reverse transcriptase inhibitor, tenofovir. Altogether, these results suggest that the kite-shaped molecules inhibit a SARS-CoV-2-specific entry step.

### 4.7. Kite-Shaped Molecules Inhibit a Post-Attachment Step

**Figure 6:**
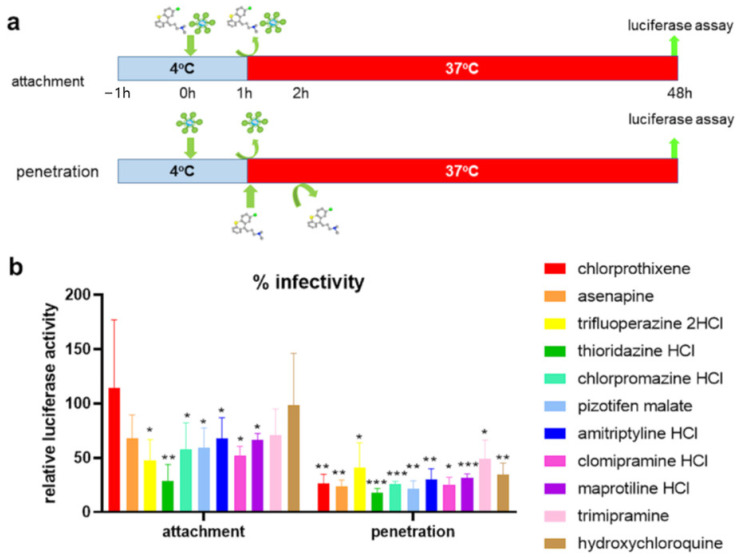
Kite-shaped molecules inhibit SARS-CoV-2 pseudovirus at post-attachment steps. Mouse leukemia virus pseudotyped with spike protein (S) from severe acute respiratory syndrome coronavirus-2 was used to infect 293T-ACE2 cells in 96-well plates for 48 h in a temperature shift experiment. (**a**) Schematic of temperature shift experiment. Cells were pre-cooled for an hour. In the attachment assay, 10 μM of drug diluted in pre-cooled virus aliquots was added to infect for an hour on ice. After 1 h, drug and virus were washed off and cells rinsed 3× with cold medium. Fresh, warm medium was added, and cells incubated for the remaining 48 h at 37 °C. In the penetration assay, pre-cooled virus without drug was added to cells at 4 °C. The virus was washed off after 1 h and cells rinsed 3× with ice-cold medium. Drug in warm medium was then added to incubate with cells for 1 h at 37 °C. The drug was then washed off with warm PBS (without Mg^2+^ and Ca^2+^) and rinsed with warm medium. Fresh, warm medium was added to continue incubation for the rest of 48 h. Addition of drug is represented by a drug image obtained from PubChem. (**b**) Infectivity was measured as luciferase activity and expressed as % infectivity to infected, own solvent control (dimethyl sulfoxide, ethanol, or water). Data are presented as mean +/− SD of four repeats for attachment assays and three repeats for penetration assays. Statistically significant differences are represented by * *p* < 0.05, ** *p* < 0.01, and *** *p* < 0.001.

To further delineate the entry step that is inhibited by the kite-shaped molecules, we undertook temperature shift experiments to distinguish between attachment and post-attachment steps that were assumed to proceed (virus attachment)—or not proceed (virus entry, post attachment)—at the low temperature (4 °C) employed in the first experiment (Figure 6a). With this assay, hydroxychloroquine did not inhibit attachment, in agreement with its main role as an in vitro lysosomotropic agent (Figure 6b). Most of the kite-shaped molecules reduced infectivity to 48–71% at the attachment step apart from chlorprothixene which did not inhibit virus attachment. Thioridazine reduced infectivity to 28% in the attachment assay. These data suggest that all the kite-shaped molecules may reduce attachment to some extent, although it should be acknowledged that this interpretation of the data depends on complete removal of the added drugs at the wash step, which may be dependent on water solubility at 4 °C (Supplementary Table S4). Overall, the data suggest that the kite-shaped molecules only modestly inhibit attachment of virus to target cells. In contrast, the kite-shaped molecules significantly reduced infectivity under conditions permissive for the post-attachment, entry step (Figure 6b), and to levels similar to that of the in vitro lysosomotropic agent, hydroxychloroquine, suggesting that the kite-shaped molecules are mainly targeting post-attachment entry. These experiments suggest that the kite-shaped molecules are mainly post-attachment entry blockers although some attachment blocking activity cannot be ruled out completely with the current assays employed.

### 4.8. Kite-Shaped Molecules Inhibit Endosomal Entry Pathway

**Figure 7:**
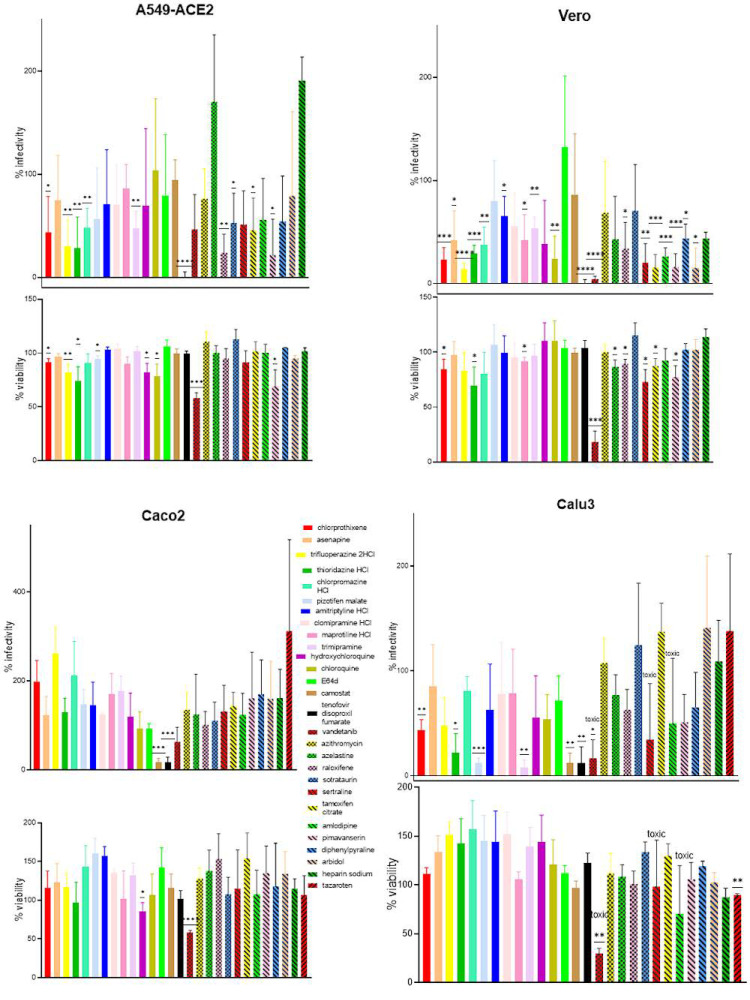
Kite-shaped molecules inhibit infectivity of SARS-CoV-2 pseudovirus in kidney and lung epithelial cells. Mouse leukemia virus pseudotyped with spike protein (S) from severe acute respiratory syndrome coronavirus-2 was used to infect A549-ACE2, Vero, Caco2, and Calu3 cells, respectively, in a 96-well plate for 48 h in the presence of drugs with 1 h pre-treatment. Infectivity was measured as luciferase activity and expressed as % infectivity to infected, own solvent control (dimethyl sulfoxide or water). Viability was measured by XTT assays in un-infected cells and expressed as % viability to solvent control (dimethyl sulfoxide or water). Data are presented as mean +/− SD of three-six repeats. Statistically significant differences are represented by * *p* < 0.05, ** *p* < 0.01, *** *p* < 0.001, and **** *p* < 0.0001.

In order to further identify the inhibitory mechanisms of the kite-shaped molecules we studied cell type specificity of the inhibition using cells with known entry pathways and compared with chemicals with known in vitro inhibitory mechanisms. As expected, tenofovir showed complete inhibition of infectivity in all cell lines tested (Figure 7). The lysosomotropic agents inhibited SARS-CoV-2 pseudovirus infection of monkey Vero but not human Calu3 and Caco2 cells. In contrast, the plasma membrane protease TMPRSS2 inhibitor, camostat mesylate, inhibited Calu3 and Caco2 but not Vero cells. This is in agreement with the utilization of endosomal fusion in Vero cells and plasm membrane fusion in Calu3 and Caco2 [8]. The kite-shaped molecules and some of the other top hits inhibited SARS-CoV-2 infection in Vero but not Caco2 cells, indicating that they probably inhibit the endosomal entry pathway. This is not surprising given that they were identified using 293T-ACE2 cells as a bait. The kite-shaped molecules showed a range of less potent inhibition in Vero cells, perhaps demonstrating some human specificity in inhibition. The lung epithelial cell line, A549, is believed to utilize the endosomal entry pathway but is reported to express a TMPRSS2 isoform that can cleave and activate the spike proteins of SARS-CoVs and MERS-CoV [53]. Surprisingly, chloroquine, hydroxychloroquine, and camostat failed to inhibit infection in A549-ACE2 cells, suggesting perhaps an alternative, non-canonical entry pathway in A549 cells. This is not impossible because the cathepsin inhibitor, E64d, inhibited 293T-ACE2 but not Vero and A549-ACE2 cells, suggesting cell-type-dependent diversification of entry mechanisms from the two main pathways. A test of ten of the kite-shaped compounds and other top hits with the A549-ACE2 system also demonstrated inhibition of SARS-CoV-2 spike-mediated infectivity in some of them. Surprisingly, some of the kite-shaped molecules inhibited infection of Calu3 cells with some potency (8% for trimipramine, 13% for Pizotifen, 22% for thioridazine, 44% for chlorprothixene) despite the reported utilization of plasma membrane fusion in Calu3 cells (and confirmed by inhibition of camostat but not chloroquine, hydroxychloroquine or E64d in this study), suggesting additional mechanisms of inhibition which are unique to the kite-shaped molecules because other top hits failed to inhibit infection of Calu3 cells [8]. Overall, these results suggest that the kite-shaped molecules target the endosomal entry pathway and may, in addition, target the plasma membrane entry pathway and an as yet unknown alternative, non-canonical pathway. Our results also suggest that both airway and kidney cells expressing ACE2 can be treated with kite-shaped inhibitors with the proviso that cell-specificity may be a factor for some of the compounds tested.

### 4.9. Kite-Shaped Molecules Show Additive, but Not Synergistic Effects with Chloroquine, Hydroxychloroquine, E64d, and/or Azithromycin on SARS-CoV-2 Pseudovirus Infection

Hydroxychloroquine has shown promise at the beginning of the pandemic but was discontinued in the WHO solidarity trial due to lack of efficacy in clinical trials [54]. We therefore sought to determine whether any of our drug hits can synergize with hydroxychloroquine to enhance drug efficacy. Since 293T-ACE2 cells showed the highest read-out, we therefore employed 293T-ACE2 as a model cell in our study of synergism. Our top hit, chlorprothixene (IC_50_ 1.94 μM) is slightly hepatotoxic (Figure 4). Therefore, we chose our second top hit, asenapine, in our synergism assay. We tested whether asenapine (IC_50_ 2.6 μM) and hydroxychloroquine (IC_50_ 0.7 μM) had synergistic actions in inhibiting SARS-CoV-2 infectivity by measuring the dose-response behavior in the assay using a matrix of concentrations of the two drugs. There was no clear indication of any synergistic effects; rather the data implied that the two drugs had additive effects on viral infectivity at low concentrations (Supplementary Information Figure S5). We also tested a mini-matrix of concentrations of asenapine in combination with E64d or azithromycin. Again, the inhibition was additive rather than synergistic (Supplementary Information Figure S6). This is consistent with the idea that both the kite-shaped molecules and E64d, a cathepsin inhibitor, block a post-attachment step. Azithromycin had been used in COVID-19 clinical trials without much benefit [55]. Our results suggest that azithromycin inhibits a post-attachment step which acts in additive to the kite-shaped molecules. Testing of three of the other top hits with chloroquine also did not show any synergistic effects. We also tested individual kite-shaped molecules and selected other top hits at 0.5 or 0.1 μM in permutation with 0.1 μM of chloroquine, E64d, and azithromycin, and again only obtained additive inhibition, suggesting that this is a common property of the kite-shaped molecules (Supplementary Information Figure S7).

## 5. Discussion

Using an MLV backbone pseudotyped with SARS-CoV-2-S, we successfully identified a class of kite-shaped molecules of TCAs and H1 receptor antagonists that have anti-viral activity and IC_50_ values in the microMolar range. Individually, a few of these had already been linked with coronavirus inhibitory activity, but this report is the first to systematically order them by activity in the FDA-approved drug toolbox and to recognize and characterize their common structural features. Chlorprothixene, one of the top hits in this study, was identified as having anti-SARS-CoV-2 activity in a repurposing study screening 8810 drugs that were either FDA-approved or investigational [56]. Methotrimeprazine and piperacetazine also emerged as top hits from that study, and these two compounds share the basic kite-shaped structure of the TCAs. Evidence from observations of patient populations has suggested there was a lower incidence of symptomatic and severe SARS-CoV-2 problems in psychiatric patients [57], and this report was followed up with an in vitro demonstration of the anti-SARS-CoV-2 activity of chlorpromazine which has subsequently entered into a clinical trial in France [58]. Some of our top hits, asenapine, thioridazine, amitriptyline, maprotiline, imipramine, and trimipramine, have been shown to inhibit native SARS-CoV-2 infection, altogether showing the robustness of our pseudotyped system in quantitative, anti-viral drug screening [21,59,60,61].

Many of the kite-shaped molecules selected are TCAs, which bind to brain-located receptors, and are currently used to treat neurological problems [62]. The IC_50_ values reported in Figure 3 may be considered modest by modern criteria [63]; for example, peak serum concentrations (Cmax) of the selected drugs within current drug treatment regimes as listed in PubChem database are in the region of 5 nM to 1–2 μM, with the highest Cmax values being for chlorprothixene (1.4 μM). Large variability in Cmax may also arise from differences in drug metabolism and clearance within patient populations [64]. The viability data are evaluated under tissue culture condition. Although clearance and intracellular and extracellular aggregation would need to factor in under in vivo condition, these FDA-approved drugs are generally non-toxic and well tolerated with ample data in the literature for drug pharmacokinetics and drug clearance from the body.

TCAs are known to bind to their neurotransmitter receptors in deep binding pockets within their transmembrane domains composed of 7 transmembrane helices, as exemplified in the 3D structures of drug/receptor complexes (4M48—nortriptyline/D2 dopamine receptor [65]; 3RZE—doxepin/H1 histamine receptor [66]). For the serotonin transporter, a similar binding site exists for citalopram (5I74) [67], a drug that lacks the central cyclic ring of the TCAs, but is otherwise very similar in its 3D structure to the kite-shaped molecules in its binding mode. A different binding site exists at an allosteric site in the pentameric Cys-loop receptor which can bind chlorpromazine (5LG3) [68]. Similarly, the binding of amitriptyline to PARP1 displays an entirely different binding site [69]. Low affinity binding of clomipramine, thioridazine and imipramine to the Ebola virus glycoprotein has also been reported, and these compounds were also shown to reduce infectivity of a pseudotyped virus system with IC_50_ values in the 8–13 μM range [70]. The binding site for these compounds does not have an equivalent in the SARS-CoV-2 spike protein; however, Ebola virus and SARS-CoV-2 may share a similar entry route into the cell [71]. Hence, none of these structural studies provided clear clues as to the likely protein target of the kite-shaped drugs for inhibition of SARS-CoV-2 infectivity, but they do demonstrate that they can bind to a variety of targets.

Perhaps of greater significance is that TCAs and similar drugs can bind and inhibit the SLC6a19 amino acid transporter [72] that is highly expressed in the intestines and kidneys [73]. The structure of LeuT, a bacterial homolog of SLC6a19 and other transporters in the SLC6a grouping has been studied in the presence of diverse tricyclic and similar antidepressants including clomipramine and nortriptyline (PDBIDs 4MMA, 4M48), revealing the nature of inhibition and the binding site [33]. SLC6a19 is known to form a stable complex with the ACE2 receptor and the SARS-CoV-2 RBD (PDBID 6M17, see also 6M18, 6M1D) [34], and residues involved in binding drugs in LeuT are conserved in the human SLC6a19 protein. Docking of clomipramine, amitriptyline, and the pharmacophore model shown in Figure 2, into the SLC6a19 atomic model was possible (Supplementary Information Figure S8) and this highlighted aromatic residues and H-bond acceptors that may be involved in the binding of the inhibitory TCAs.

Although the kite-shaped molecules generally share a common mode of action, they may also possess unique mechanisms of inhibition. Whereas most of the kite-shaped molecules displayed a similar inhibitory pattern of infectivity, chlorprothixene and chlorpromazine showed some discrepancies. Chlorpromazine has been known to inhibit clathrin-mediated endocytosis, which is utilized by the VSV to enter cells, so it is not surprising that it will inhibit VSV-G pseudovirus infection to some extent [74,75]. Imipramine (also one of our top hits), a parent compound of trimipramine, blocks macropinocytosis-a potential route of viral endocytosis although activity has not been demonstrated in SARS-CoV-2 infection [76]. Three of our top hits, amitriptyline, maprotiline, and imipramine, have been shown to prevent SARS-CoV-2 infection by inhibiting acid sphingomyelinase, placing them in a class of host-targeting agents [60]. We currently do not have enough evidence to propose whether our kite-shaped drug hits are direct-acting antivirals and/or host-targeting agents. Further work will be required to identify the common and unique modes of action of our drug hits in order to facilitate the formulation of a drug cocktail.

Although it is well recognized that SARS-CoV-2 infects lungs, gut, and eyes, increasing evidence suggest liver and kidney tropism, with the kidney predicted to be the most susceptible [77]. Hence, the kidney cell line we used initially in this study is relevant to SARS-CoV-2 infection biology. 293T cells are devoid of TMPRSS2, hence unable to trigger plasma membrane fusion [14]. As a result, our anti-viral screening is limited to drug hits that inhibit the endosomal entry pathway. However, our results illustrate that the kite-shaped molecules may be more versatile than just blocking the endosomal entry pathway as some of them showed modest potency in A549-ACE2 cells (which presumably employs a non-canonical entry mechanism) and good potency in Calu3 cells (which employs plasma membrane fusion), making them promising candidates as universal entry blockers. It is of paramount importance for an anti-viral regime to be able to target both pathways. Selective targeting of the default membrane fusion pathway may drive the evolution of SARS-CoV-2 into the embrace of the endosomal fusion pathway.

In conclusion, our study has generated a class of kite-shaped molecules that target a potentially conserved post-attachment step of SARS-CoV-2 cell entry which could inform clinical trials in the current crisis. We have also created a pharmacophore that will allow for improvement in drug design as a broad-spectrum antiviral for future pandemics.

## Figures and Tables

**Table 1:** A list of top hits with ≥65% VSV-G infectivity in a negative screen.

		% Infectivity of ^a^ SARS-2-Spike pv	% Viability of SARS-2-Spike pv	% Infectivity of ^b^ VSV-G pv	Targets
chloroquine diphosphate 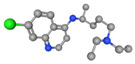		1	123	83	autophagy/ubiquitination, endosomal fusion, receptor, anti-malarial
vandetanib 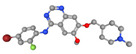		1	89	125	tyrosine kinase ^f^ VEGFR/EGFR
hydroxychloroquine sulfate 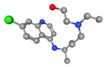		2	90	103	autophagy/ubiquitination, endosomal fusion, receptor, inflammation
azithromycin 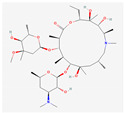		3	100	93	macrolide antibiotic 50S ribosomal subunit protein synthesis
pimavanserin 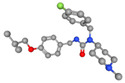		3	70	72	^g^ 5-HT receptor ^h^ GPCR/G protein
azelastine HCl 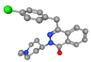	kite-like	4	98	81	histamine H1 receptor
chlorprothixene 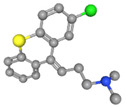	kite	4	97	^e^ 48 (30–66)	5-HT2, dopamine D1, D2, D3, histamine H1, muscarinic, α1 adrenergic receptors
azithromycin dihydrate 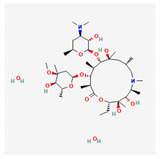		4	94	100	macrolide antibiotic 50S ribosomal subunit protein synthesis
asenapine 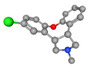	kite	4	92	^e^ 71 (71–71)	5-HT, adrenergic receptors
raloxifene HCl 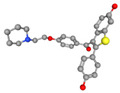	kite-like	5	92	65	estrogen/progestogen receptor
trifluoperazine 2HCl 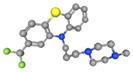	kite	5	83	^e^ 80 (80–80)	dopamine D2 receptor autophagy/ubiquitination
^c^ Timosaponin A3 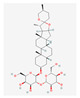		5	55	85	cancer
thioridazine HCl 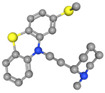	kite	6	71	^e^ 69 (64–73)	dopamine D2; serotonin 5-HT2 receptors calcium channel protein
chlorpromazine HCl 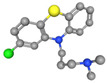	kite	6	65	^e^ 58 (46–71)	dopamine receptor
pizotifen malate 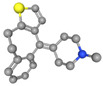	kite	7	106	^e^ 77 (70–84)	5-HT2, D2 receptors inflammation
sertraline HCl 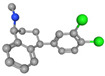		8	95	71	^i^ SSRIs
sotrastaurin 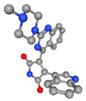		8	89	76	^j^ PKC/TGF-β/Smad signaling
clomipramine HCl 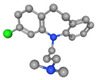	kite	9	115	^e^ 78 (70–86)	5-HT receptor ^k^ SERT/NET dopamine transporter
amitriptyline HCl 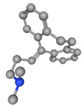	kite	9	112	^e^ 80 (71–88)	5-HT4/5-HT2; serotonin/norepinephrine receptors
cyclobenzaprine HCl 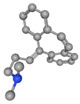	kite	9	101	81	5-HT2 receptor GPCR/G protein
maprotiline HCl 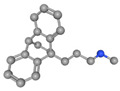	kite	9	99	89	5-HT receptor GPCR/G protein
E64d 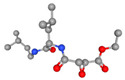		9	95	105	cathepsin cysteine protease
tamoxifen citrate 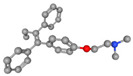		10	107	81	estrogen/progestogen receptor
desloratadine 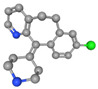	kite	10	99	82	histamine H1 receptor
benztropine mesylate 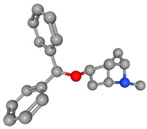	kite-like	10	96	93	histamine receptor, a central muscarinic antagonist, inhibits dopamine uptake
promethazine HCl 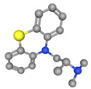	kite	11	99	107	peripheral H1 receptors; central histaminergic receptors
amlodipine 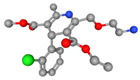		12	115	78	calcium channel P-glycoprotein
solifenacin succinate 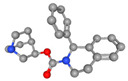		12	99	86	M3 muscarinic receptor
trimipramine maleate 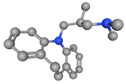	kite	14	92	^e^ 95 (83–108)	histamine H1 receptor
diphenylpyraline HCl 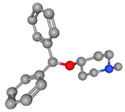		15	106	108	antihistamine, dopamine reuptake inhibitor
imipramine 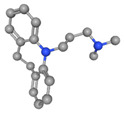	kite	16	99	84	5-HT, H1 receptors; serotonin and norepinephrine transporters
prochlorperazine 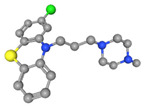	kite	16	94	80	dopamine D2 receptor
cyproheptadine HCl 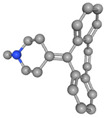	kite	17	94	92	histamine receptor; serotonin and histamine antagonist; antimuscarinic
amlodipine besylate 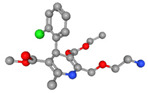		19	116	100	L-type calcium channel P-glycoprotein
anidulafungin 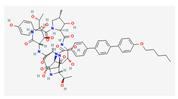		19	109	125	antifungal 1,3 beta-D glucan synthase
amoxapine 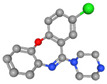	kite	20	103	95	^l^ GLYT2a transport activity
^d^ tazarotene 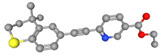		225	107	102	^m^ RARs; topical retinoid; antiproliferative; inducer of ^n^ TIG3 tumor suppressor

Ball and stick images are from PubChem 3D conformer visualization. Blue = N; red = O; green = Cl (Fl in vandetanib, pimavanserin and trifluoperazine); yellow = S; brown = bromide. Azithromycin, timosaponin A3, and anidulafungin have less defined structures. ^a^ SARS-2-spike pv severe acute respiratory syndrome coronavirus 2 spike protein pseudovirus. ^b^ VSV-G pv vesicular stomatitis virus glycoprotein pseudovirus. ^c^ natural compound. ^d^ tazarotene a potentiator hit. ^e^ mean (range) of two repeats. ^f^ VEGFR/EGFR vascular endothelial growth factor receptor/epidermal growth factor receptor. ^g^ 5-HT receptors 5-hydroxytryptamine receptors, or serotonin receptors. ^h^ GPCR/G protein G protein-coupled receptors. ^i^ SSRIs selective serotonin reuptake inhibitors. ^j^ PKC/TGF-β protein kinase C/transforming growth factor-beta. ^k^ ERT/NET serotonin norepinephrine transporter. ^l^ GLYT2a neuronal and glial glycine transporter 2a. ^m^ RARs retinoid acid receptors. ^n^ TIG3 tazarotene-induced gene 3.

## Data Availability

The pharmacophore and docking results may be obtained from the corresponding author.

## Supplementary Materials

The following are available online at https://www.mdpi.com/article/10.3390/v13112306/s1, Figure S1: SARS-CoV-2 pseudotyped virus in anti-viral drug screening. Figure S2: Infectivity of pseudoviruses in a range of cell types. Figure S3: Quantification of ACE2 and spike protein. Figure S4: Dose-response curves of top hits in SARS-CoV-2-S inhibition. Figure S5: Asenapine and hydroxychloroquine show additive inhibitory effect on SARS-CoV-2 infectivity. Figure S6: Drug hits show additive inhibitory effect on SARS-CoV-2 infectivity. Figure S7: Drug hits show additive inhibitory effect on SARS-CoV-2 infectivity. Figure S8: diagrammatic representation of ligand-protein interaction and pharmacophore mapping. Table S1: Kinases with similar structures to vandetanib are false positives. Table S2: Oestrogen receptor modulators with similar structures are a hit and a false positive. Table S3: Kite-shaped molecules in order of inhibition of pseudovirus infectivity. Table S4: Water solubility of kite-shaped molecules and hydroxychloroquine.
