# Supplementary material for: Kite-Shaped Molecules Block SARS-CoV-2 Cell Entry at a Post-Attachment Step
Source: Viruses. 2021 Nov 19;13(11):2306. doi: 10.3390/v13112306 (PMC8619434; doi:10.3390/v13112306)
Supplement: Supplementary file 1 [file viruses-13-02306-s001.zip › viruses-1401147-supplementary.pdf]

Kite-shaped molecules block SARS-CoV-2 cell entry at a post-attachment step

Running title: Repurposing for coronaviruses.

Shiu-Wan Chan*, Talha Shafi and Robert C. Ford

Faculty of Biology, Medicine and Health, School of Biological Sciences, The University of Manchester, Michael Smith Building, Oxford Road, Manchester M13 9PT, United Kingdom

Supplementary information

Table S1

Table S2

Table S3

Table S4

Figure S1

Figure S2

Figure S3

Figure S4

Figure S5

Figure S6

Figure S7

Figure S8

Table S1. Kinases with similar structures to vandetanib are false positives

		% infectivity of [a]SARS-2-spike pv	% viability of SARS-2-spike pv	% infectivity of [b]VSV-G pv	Targets
vandetanib	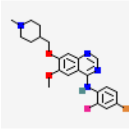	1	89	125	tyrosine kinase<br>[c]VEGFR/EGFR
cediranib (AZD217)	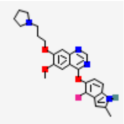	5	85	42	VEGFR
MLN8237 (Alisertib)	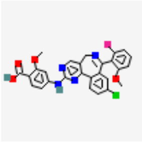	15-18	63-83	18	Aurora kinase inhibitor<br>(serine/threonine protein kinase)
Foretinib (GSK1363089)	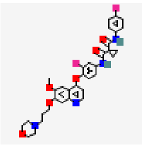	10	63	11	VEGFR and hepatocyte growth factor receptor
Axitinib (AG 013736)	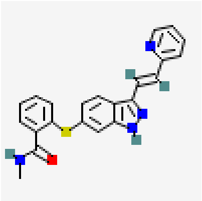	1	69	2	VEGFR1/c-Kit inhibitor
LY2784544	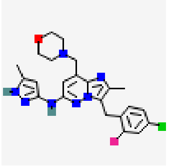	2	76	1	Janus kinase 2
GS-9973	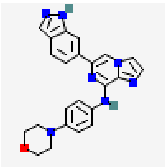	0	69	1	spleen tyrosine kinase
dinaciclib	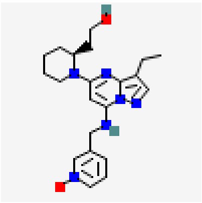	0	55	1	cyclin-dependent kinases

[a]SARS-2-spike pv   severe acute respiratory syndrome coronavirus 2 spike protein pseudovirus

[b]VSV-G pv   vesicular stomatitis virus glycoprotein pseudovirus

[c]VEGFR/EGFR vascular endothelial growth factor receptor/ epidermal growth factor receptor

drug structures obtained from PubChem

Table S2. Oestrogen receptor modulators with similar structures are a hit and a false positive

			% infectivity of [a]SARS-2-spike pv	% viability of SARS-2-spike pv	% infectivity of [b]VSV-G pv
bazedoxifene Hcl	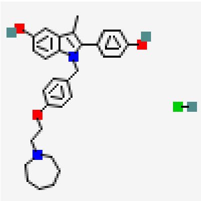	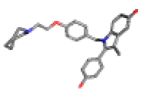	3	88	13
raloxifene HCl	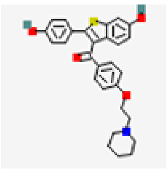	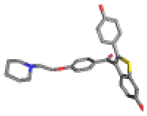	5	92	65

[a]SARS-2-spike pv   severe acute respiratory syndrome coronavirus 2 spike protein pseudovirus

[b]VSV-G pv   vesicular stomatitis virus glycoprotein pseudovirus

drug structures obtained from PubChem

Table S3. Kite-shaped molecules in order of inhibition of pseudovirus infectivity.

	% infectivity[a]	% viability[b]	2D structure	3D structure
chlorprothixene	4.1	97	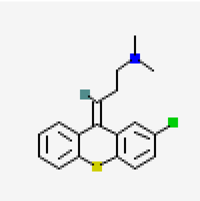	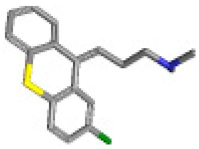

asenapine	4.38	92	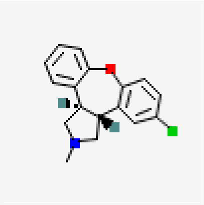	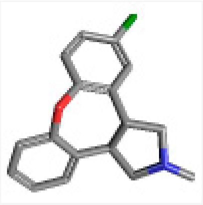
Trifluoperazine 2HCl	4.84	83	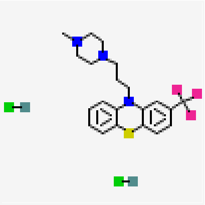	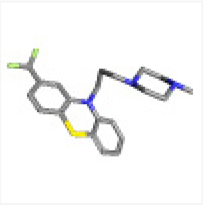
thioridazine HCL	5.98	71	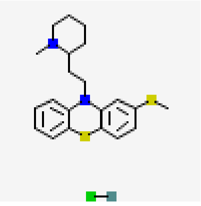	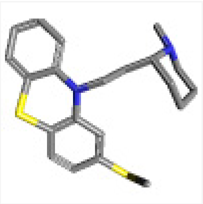
Chlorpromazine HCl	6.24	65	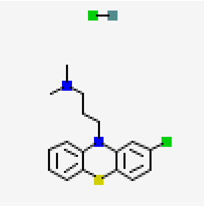	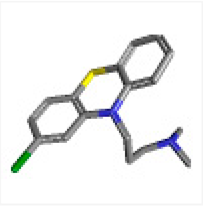
Pizotifen Malate	7.01	106	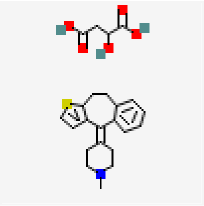	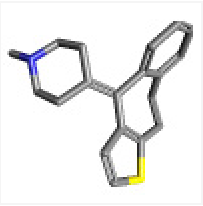
Amitriptyline HCl	9.05	112	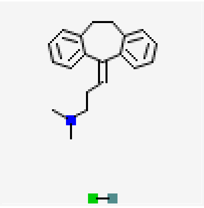	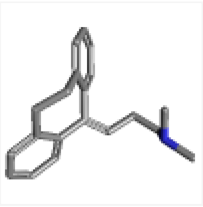
Clomipramine HCl	9.23	115	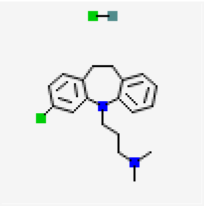	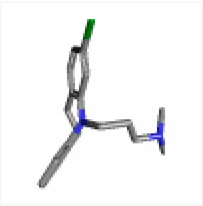

maprotiline hcl	9.38	99	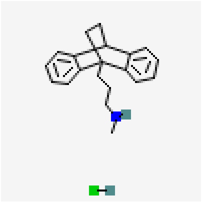	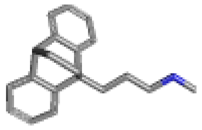
cyclobenzaprine HCl	9.49	101	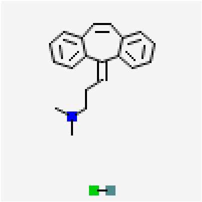	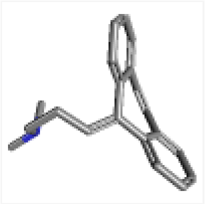
Desloratadine	10.25	99	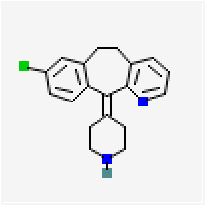	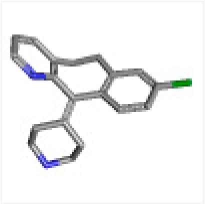
promethazine HCl	11.32	99	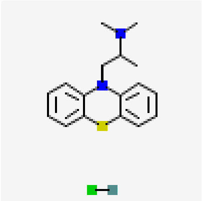	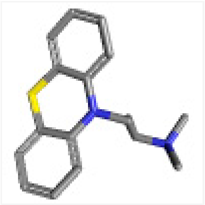
Carvedilol	13.1	81	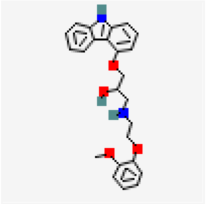	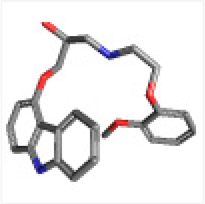
trimipramine	13.54	92	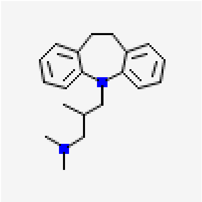	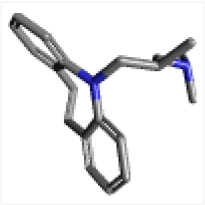
MLN8237	15.36	63	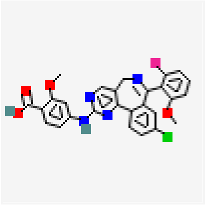	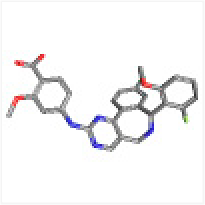

S/GSK1349572	16.2	94	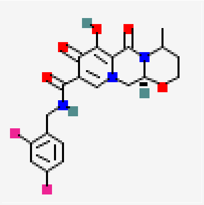	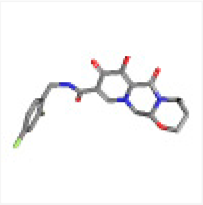
Imipramine-	16.34	99	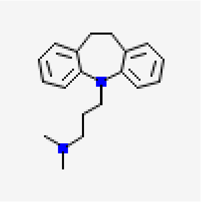	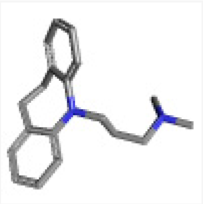
Prochlorperazine	16.41	94	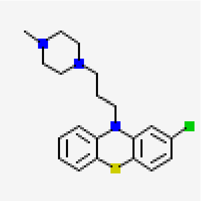	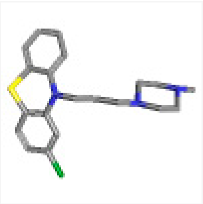
Cyproheptadine hydrochloride	16.9	94	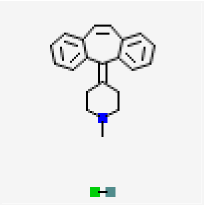	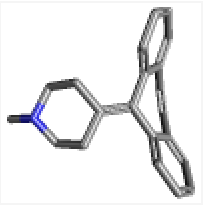
amoxapine	19.9	103	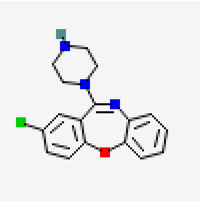	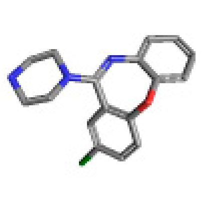
Olanzapine	26.22	106	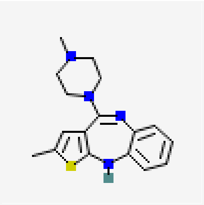	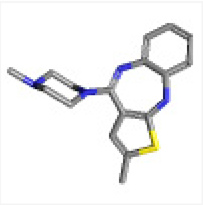
elacridar	27.04	97	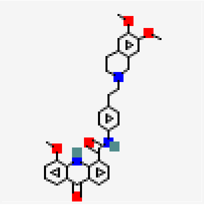	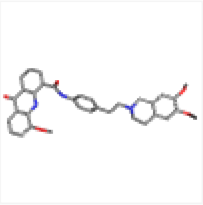

Clozapine	28.31	103	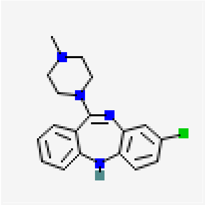	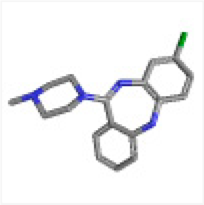
Loxapine Succinate	35.01	94	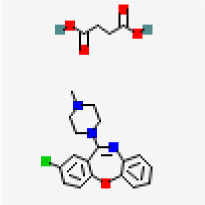	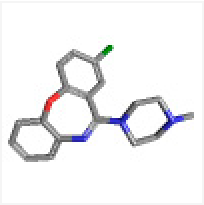
Dehydrocostus Lactone	36.23	90	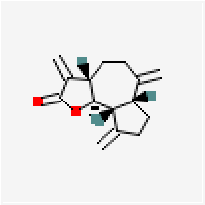	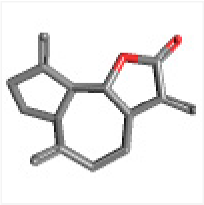
Rupatadine Fumarate	38.01	74	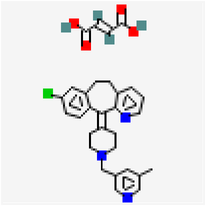	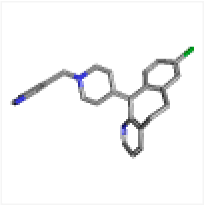
mianserin Hcl	41.95	100	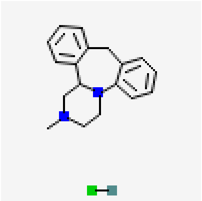	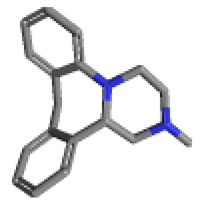
Clofazimine	45.61	77	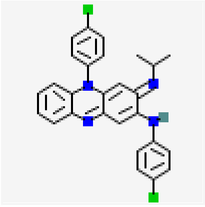	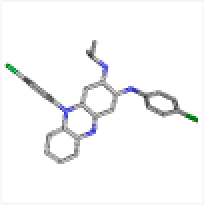
Mesoridazine besylate	48.73	97	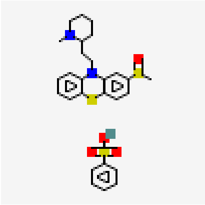	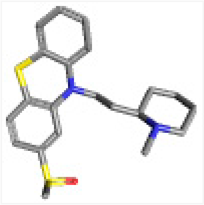

conivaptan hcl	51.97	102	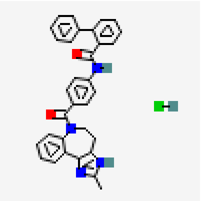	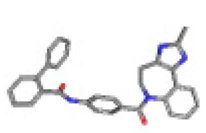
Tacrine hydrochloride	57.04	97	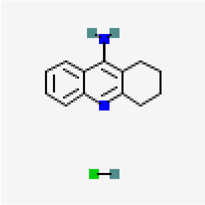	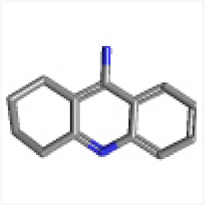
Ketotifen Fumarate	60.4	97	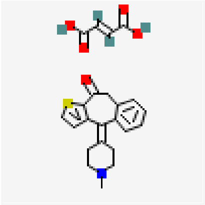	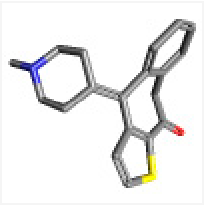
lonafarnib	64.73	111	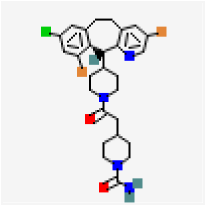	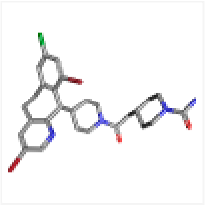
loratadine	67.15	94	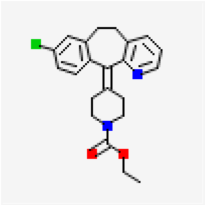	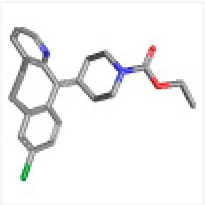
Diphenhydramine HCl	67.66	101	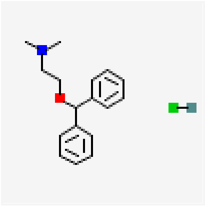	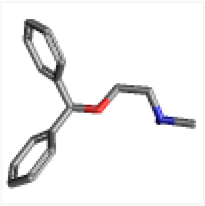
Mirtazapine	72.63	104	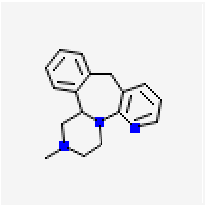	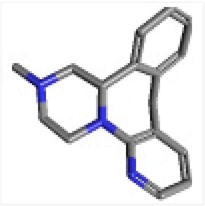

Epinastine HCl	72.78	94	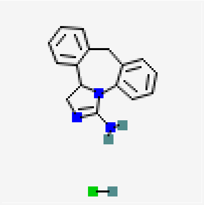	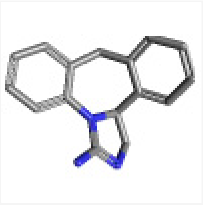
Etodolac	76.15	100	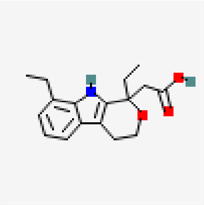	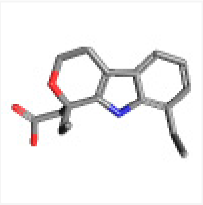
Carprofen	80.32	103	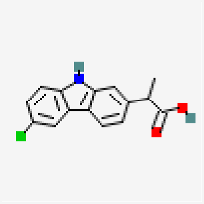	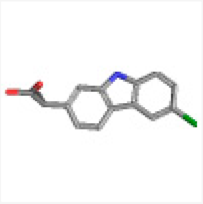
Nevirapine	80.71	103	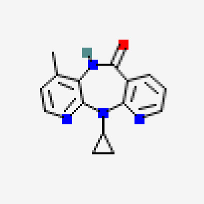	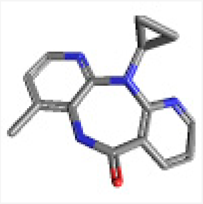
Carbamazepine	81.27	105	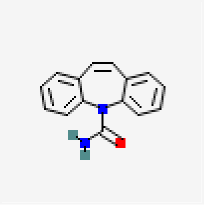	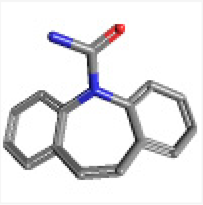
Diacerein	81.89	126	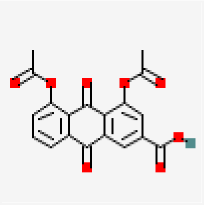	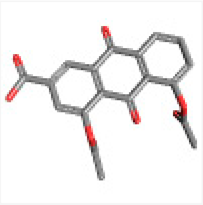
Alcaftadine	85.44	97	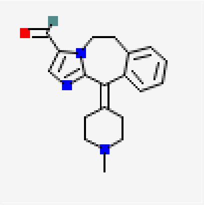	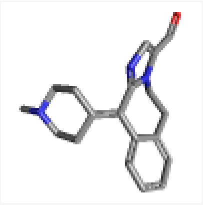

Pranoprofen	87.46	114	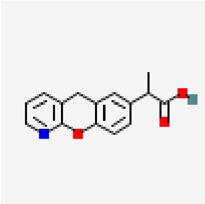	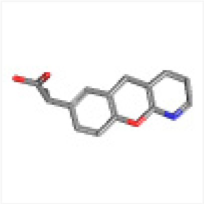
Eslicarbazepine acetate	90.75	102	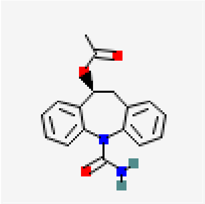	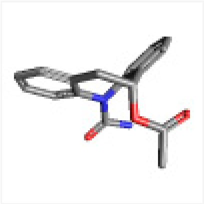
Olopatadine HCl	91.21	99	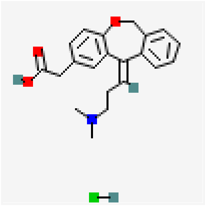	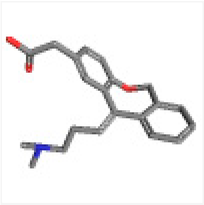
Dibenzothiophen e	92.11	102	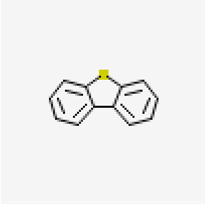	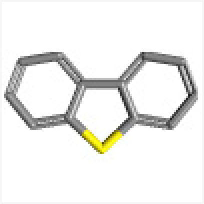
Tianeptine sodium	94.8	102	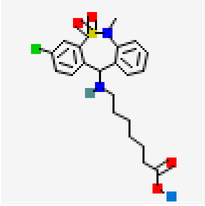	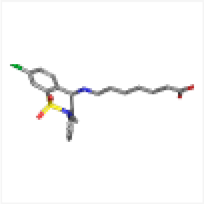
Oxcarbazepine	95.77	109	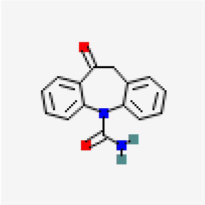	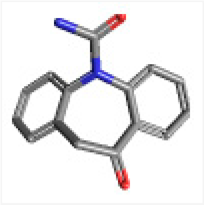
flumazenil	99.18	104	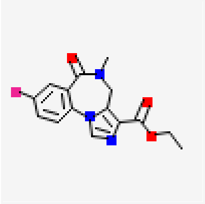	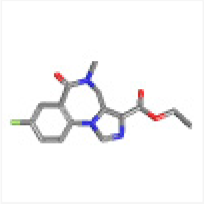

Quetiapine Fumarate	99.77	96	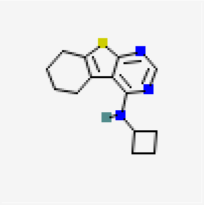	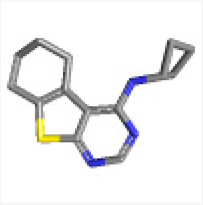
8-methoxypsoralen	100.26	105	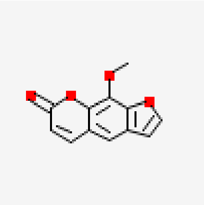	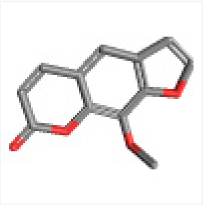
Ramelteon	104.18	99	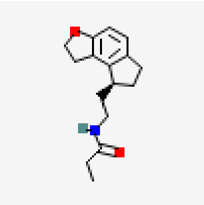	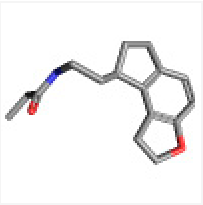
Aloin	107.91	103	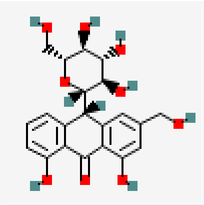	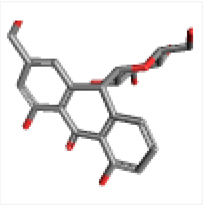
5-methoxypsoralen	109.79	111	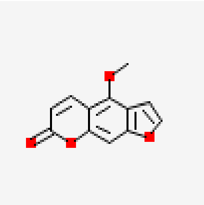	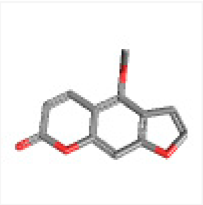
quetiapine	112.56	101	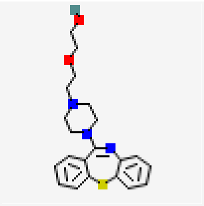	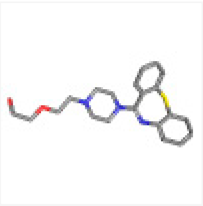

a % infectivity relative to the infected, DMSO control

b % viability relative to the un-infected, DMSO control

drug structures obtained from PubChem

Table S4. Water solubility of kite-shaped molecules and hydroxychloroquine

Drug	water solubility[a]	water solubility[b]
hydroxychloroquine sulphate	60 µM	200mM
chlorprothixene HCl	0.9 µM	28mM
asenapine maleate	78 µM	9mM
trifluoperazine HCl	18 µM	104mM
thioridazine HCl	2.1 µM	122mM
chlorpromazine HCl	12 µM	141mM
pizotifen malate	16 µM	0.3mM[c]
amitriptyline HCl	14 µM	99mM
clomipramine HCl	41 µM	71mM
maprotiline HCl	150 µM	159mM
trimipramine maleate	88 µM	5mM

a Values are predictions for the uncharged form obtained from https://go.drugbank.com

b. Values are for the salts and from chemical supplier datasheets, where available.

c. In a 1:8 dimethylformamide:water mixture.

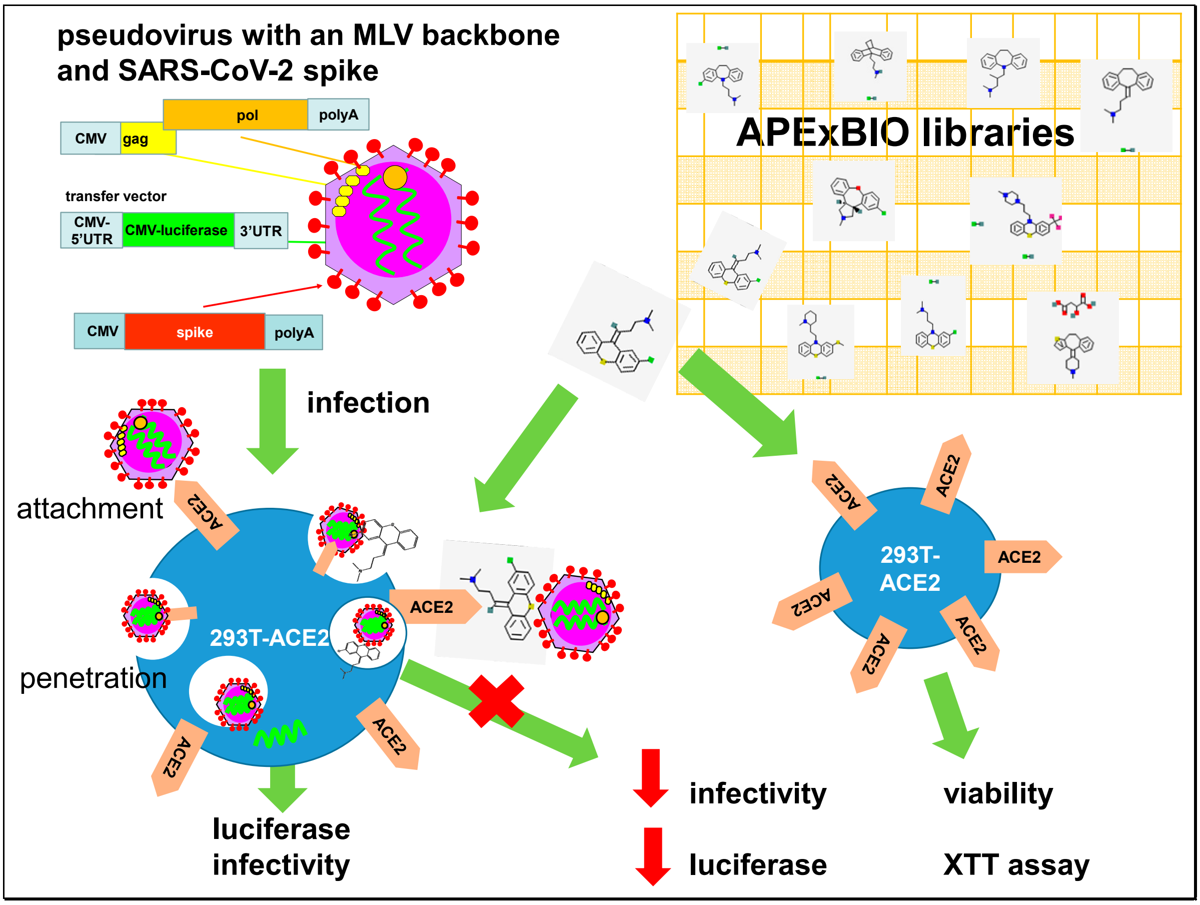

Figure S1. SARS-CoV-2 pseudotyped virus in anti-viral drug screening.

Pseudovirus was generated on a mouse leukaemia virus (MLV) backbone using a three-plasmid system consisting of an expression vector for MLV *gag* and *pol*, a transfer vector carrying a luciferase reporter gene and an expression vector encoding the SARS-CoV-2 spike protein. Pseudovirus was used to infect 293T cells stably expressing the human angiotensin-converting enzyme 2 (ACE2). Pseudovirus entry was mediated by the binding of the spike protein to the ACE2 which then undergoes receptor-mediated endocytosis to trigger endosomal fusion to release the luciferase reporter gene into cell cytoplasm. Infectivity was measured as luciferase read-out. Drugs from two APExBIO libraries were screened for their ability to inhibit infectivity by measuring the reduction in luciferase activity. Since the spike protein only mediates virus entry, the pseudovirus system could be used to identify drug hits that inhibit SARS-CoV-2 entry steps only. Drug cytotoxicity was measured using an XTT viability assay in non-infected cells. Drug images were obtained from PubChem and

APExBIO.

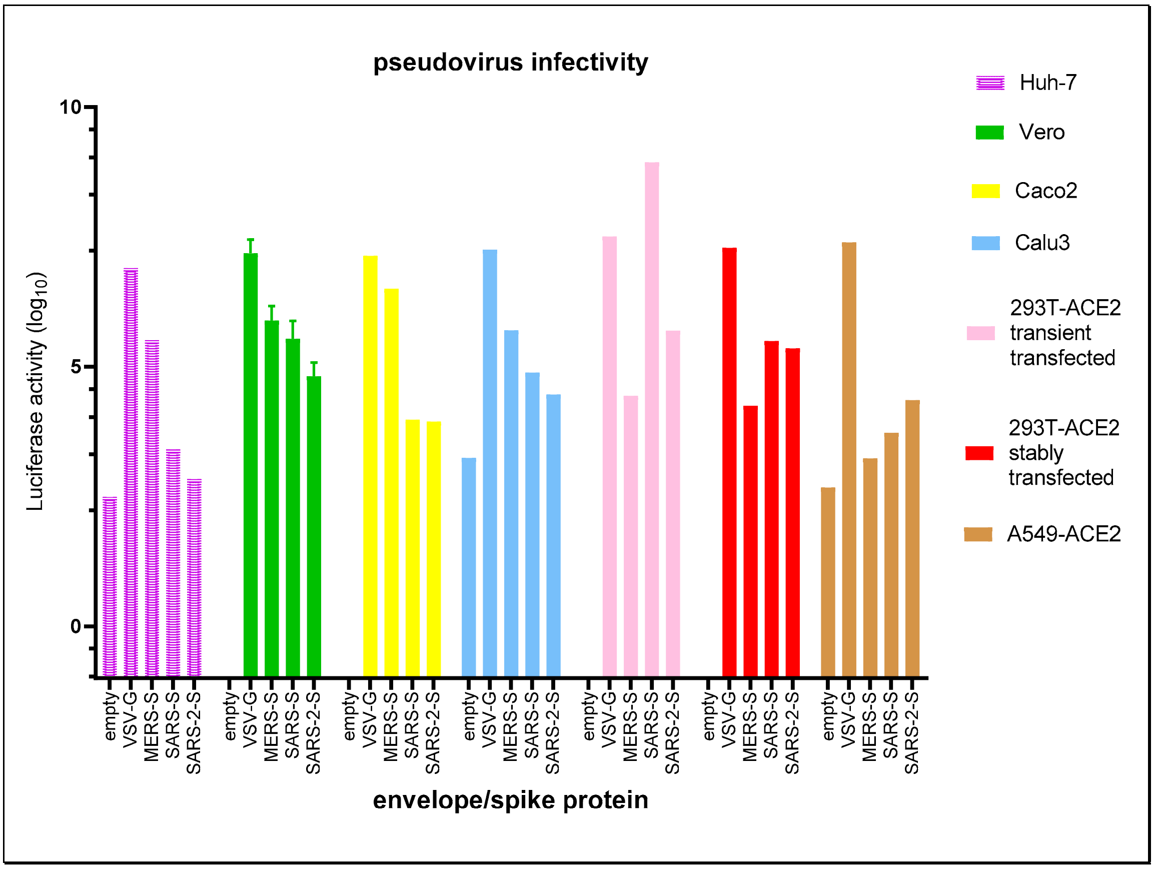

Figure S2. Infectivity of pseudoviruses in a range of cell types.

Mouse leukaemia virus pseudotyped with no envelope protein (empty), glycoprotein from vesicular stomatitis virus (VSV-G) and spike protein (S) from Middle East respiratory syndrome coronavirus (MERS-S), severe acute respiratory syndrome coronavirus (SARS-S) and SARS-2-S was used to infect a range of cell types, as indicated, in 24-well plates for 72h. Infectivity was measured as luciferase activity. Data represent the mean of two repeats for Vero cells and one experimental result for the other cell types.

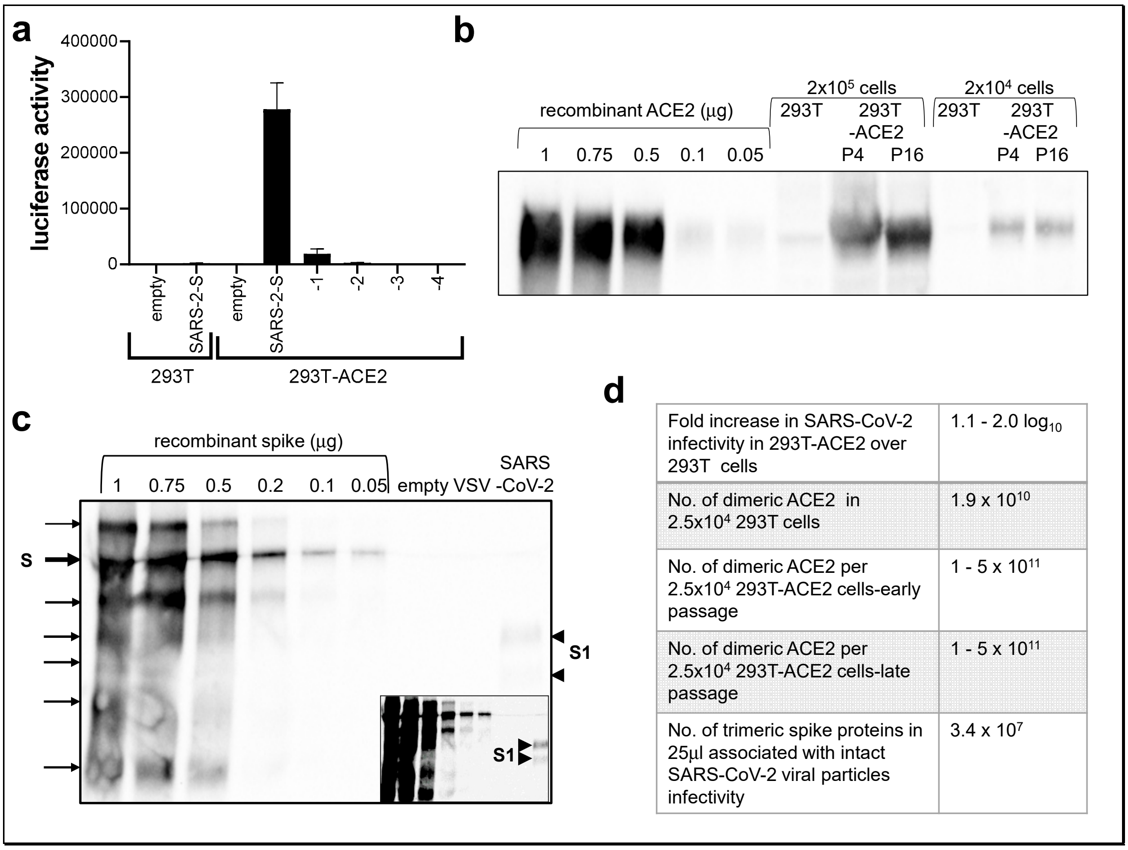

Fold increase in SARS-CoV-2 infectivity in 293T-ACE2 over 293T cells	1.1 - 2.0 $\log_{10}$
No. of dimeric ACE2 in $2.5x10^4$ 293T cells	1.9×10^{10}
No. of dimeric ACE2 per $2.5x10^4$ 293T-ACE2 cells-early passage	$1 - 5 \times 10^{11}$
No. of dimeric ACE2 per $2.5x10^4$ 293T-ACE2 cells-late passage	$1 - 5 \times 10^{11}$
No. of trimeric spike proteins in 25μl associated with intact SARS-CoV-2 viral particles infectivity	3.4×10^7

Figure S3. Quantification of ACE2 and spike protein.

(a) Serial ten-fold dilutions of the SARS-CoV-2-S pseudotyped virus were used to infect 293T-ACE2 cells. Luciferase activity was measured at 72h and compared to that of 293T cells infected with undiluted pseudovirus. Data are presented as mean +/- SD of two repeats. (b) Western blot of ACE2 from $2.5x10^5$ and $2.5x10^4$ 293T cells and 293T-ACE2 cells from early (P4) and late (P16) passages. The protein bands were quantified against a standard curve of recombinant ACE2. (c) Western blot of spike protein from 10μl of empty, VSV-G and SARS-CoV-2-S pseudovirus particles. The protein bands were quantified against a standard curve of recombinant spike protein. The spike protein in the SARS-CoV-2-S pseudovirus has been cleaved to yield the S1 subunit. The inset shows the same blot at higher contrast for clarity. Low exposure blot was used in quantification. The recombinant protein is near full-length and has been stabilized with the removal of the furin-cleavage site and exhibits many glycosylated and degraded forms. (d) A table summarizing the

calculations. After estimating the μg of ACE2/spike proteins from the standard curve, the number of molecules was calculated by converting μg into moles multiplied by Avogadro's number. The range reflects data calculated from $2.5x10^5$ and $2.5x10^4$ cell loading. The number of spike proteins was adjusted using the assumption that 76% of the spike protein are not associated with viral particles (they are secreted or degraded virion associated with extracellular vesicles). Amongst the viral particles, 80% are empty viral particles (non-infectious, no genome but retain spike) and the rest of the 20% intact particles had 0.4% infectivity (1).

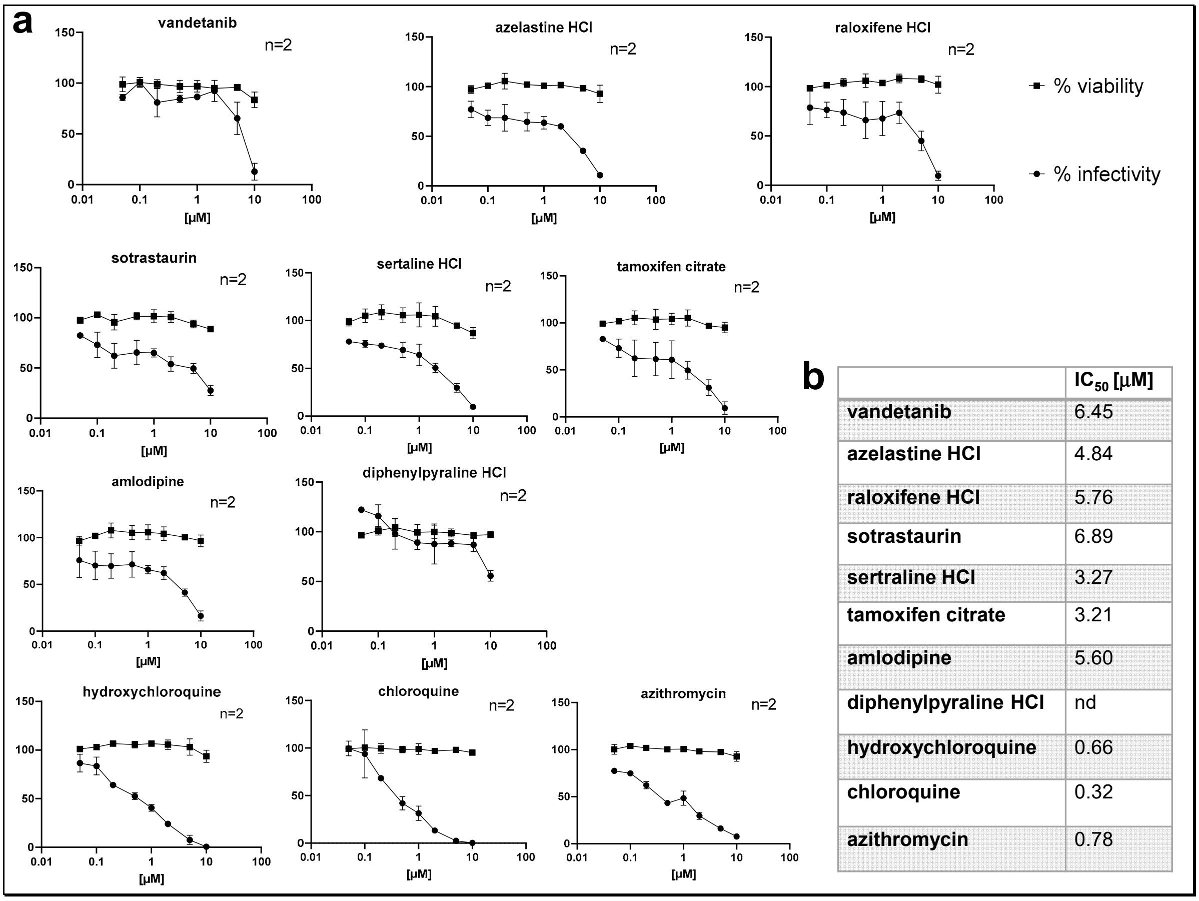

	IC_{50} [μM]
vandetanib	6.45
azelastine HCl	4.84
raloxifene HCl	5.76
sotrastaurin	6.89
sertraline HCl	3.27
tamoxifen citrate	3.21
amlodipine	5.60
diphenylpyraline HCl	nd
hydroxychloroquine	0.66
chloroquine	0.32
azithromycin	0.78

Figure S4. Dose-response curves of top hits in SARS-CoV-2-S inhibition. Mouse leukaemia virus pseudotyped with spike protein (S) from severe acute respiratory syndrome coronavirus-2 was used to infect 293T-ACE2 cells in 96-well plates for 48h in the presence of serial doses of the drug, as indicated, with 1h pre-treatment. (a) Infectivity was measured

as luciferase activity and expressed as % infectivity to infected, own solvent control (dimethylsulphoxide or water). Viability was measured by XTT assays in un-infected cells and expressed as % viability to un-infected solvent control (dimethylsulphoxide or water). Data are presented as mean +/- SD of two repeats. The square symbols are for cell viability (% of control) and the round symbols are for infectivity by the pseudovirus (% of control). (b) Summary of IC_{50} values. nd=not determined.

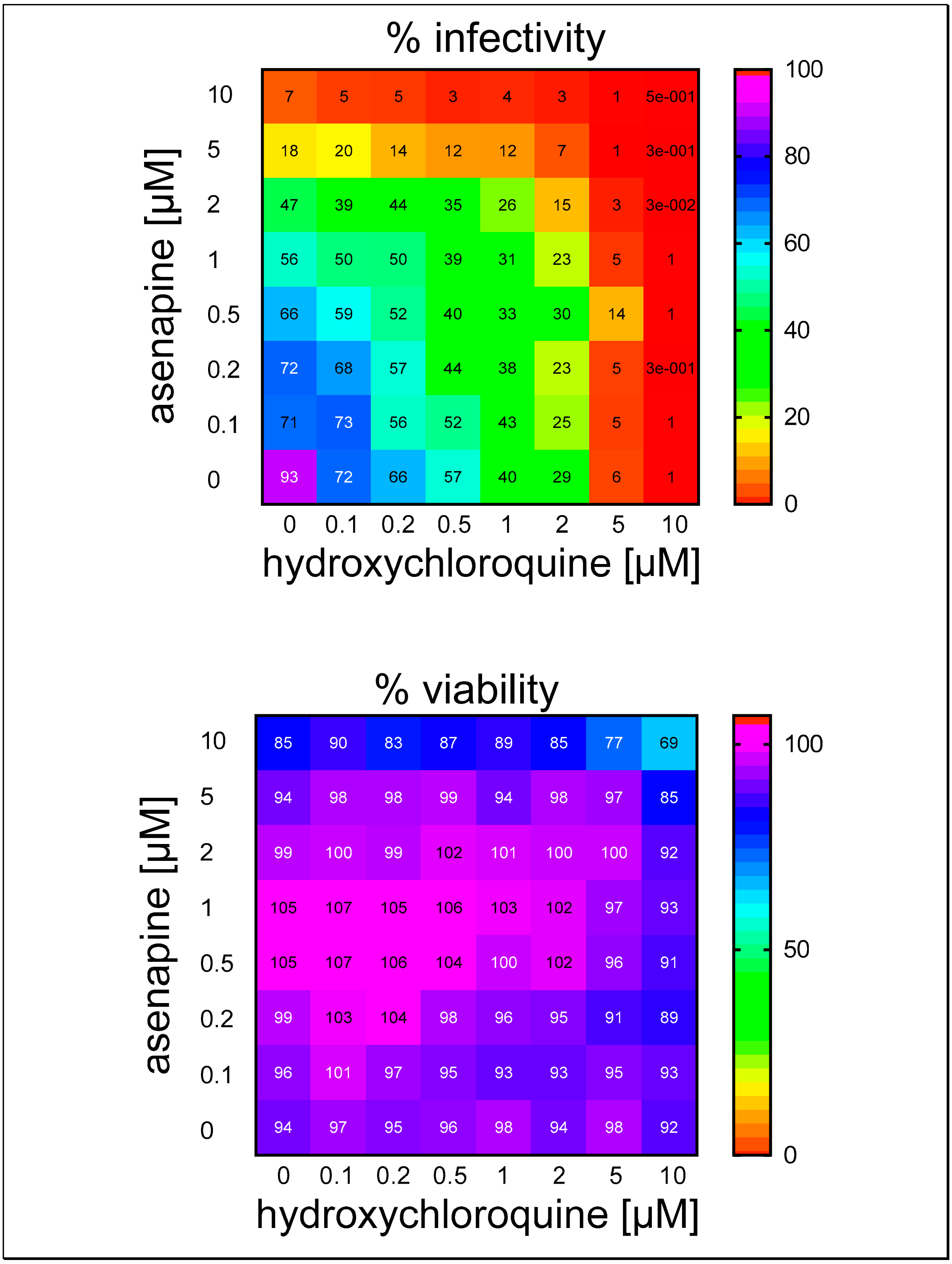

Figure S5. Asenapine and hydroxychloroquine show additive inhibitory effect on SARS-CoV-2 infectivity. Mouse leukaemia virus pseudotyped with spike protein (S) from severe acute respiratory syndrome coronavirus-2 was used to infect 293T-ACE2 cells in 96-well plate for 48h in the presence of serial doses of the asenapine and hydroxychloroquine, as indicated, with 1h pre-treatment. Infectivity was measured as luciferase activity and

expressed as % infectivity to infected, own solvent control (dimethylsulphoxide or water). Viability was measured by XTT assays in un-infected cells and expressed as % viability to un-infected solvent control (dimethylsulphoxide or water). Date are presented as heat maps generated using Prism9.0 (GraphPad). Data are from one repeat.

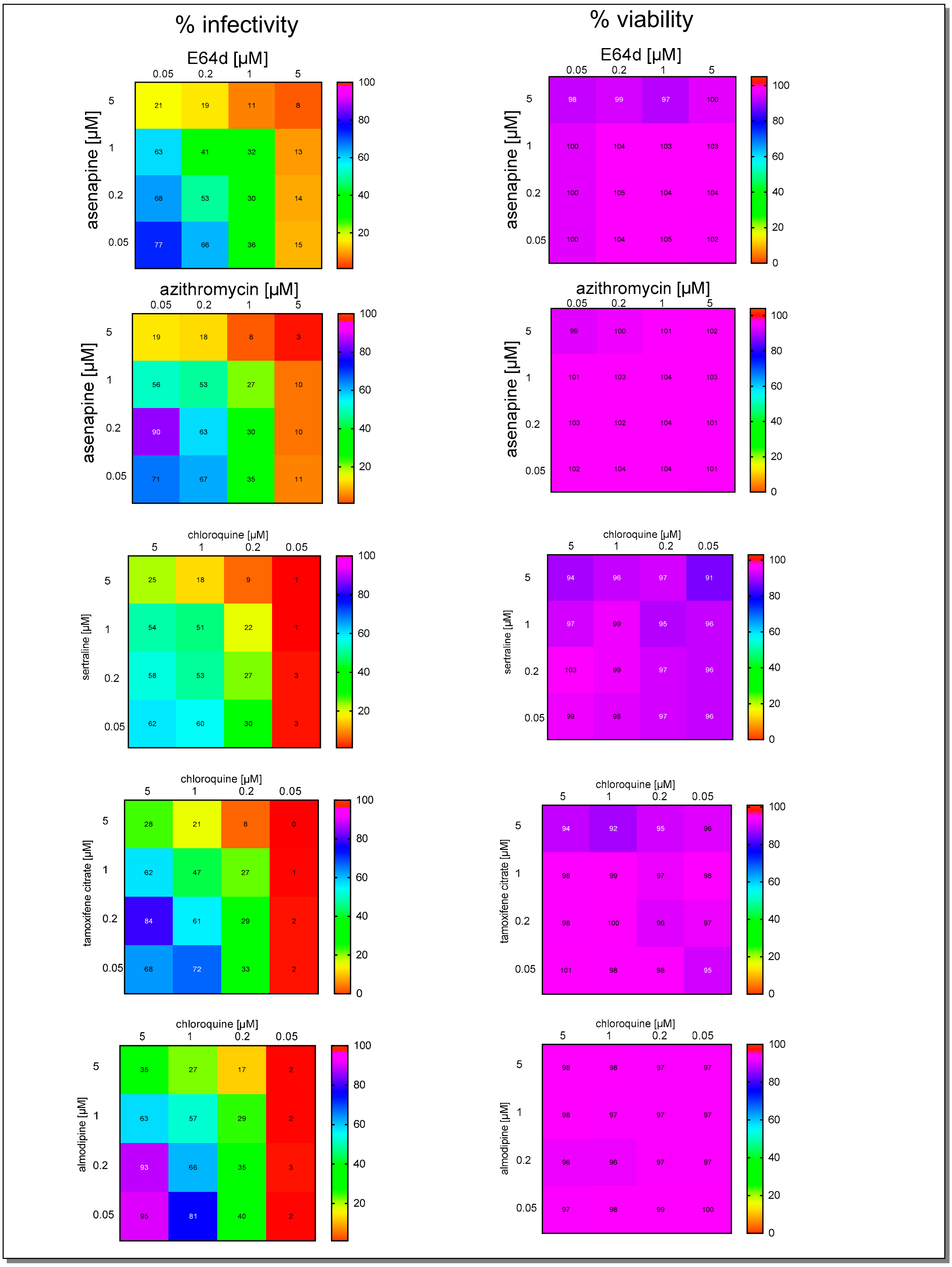

Figure S6. Drug hits show additive inhibitory effect on SARS-CoV-2 infectivity. Mouse leukaemia virus pseudotyped with spike protein (S) from severe acute respiratory syndrome coronavirus-2 was used to infect 293T-ACE2 cells in 96-well plate for 48h in the presence of

serial doses of the drug combination, as indicated, with 1h pre-treatment. Infectivity was measured as luciferase activity and expressed as % infectivity to infected, own solvent control (dimethylsulphoxide or water). Viability was measured by XTT assays in un-infected cells and expressed as % viability to un-infected solvent control (dimethylsulphoxide or water). Date are presented as heat maps generated using Prism9.0 (GraphPad). Data are from one repeat.

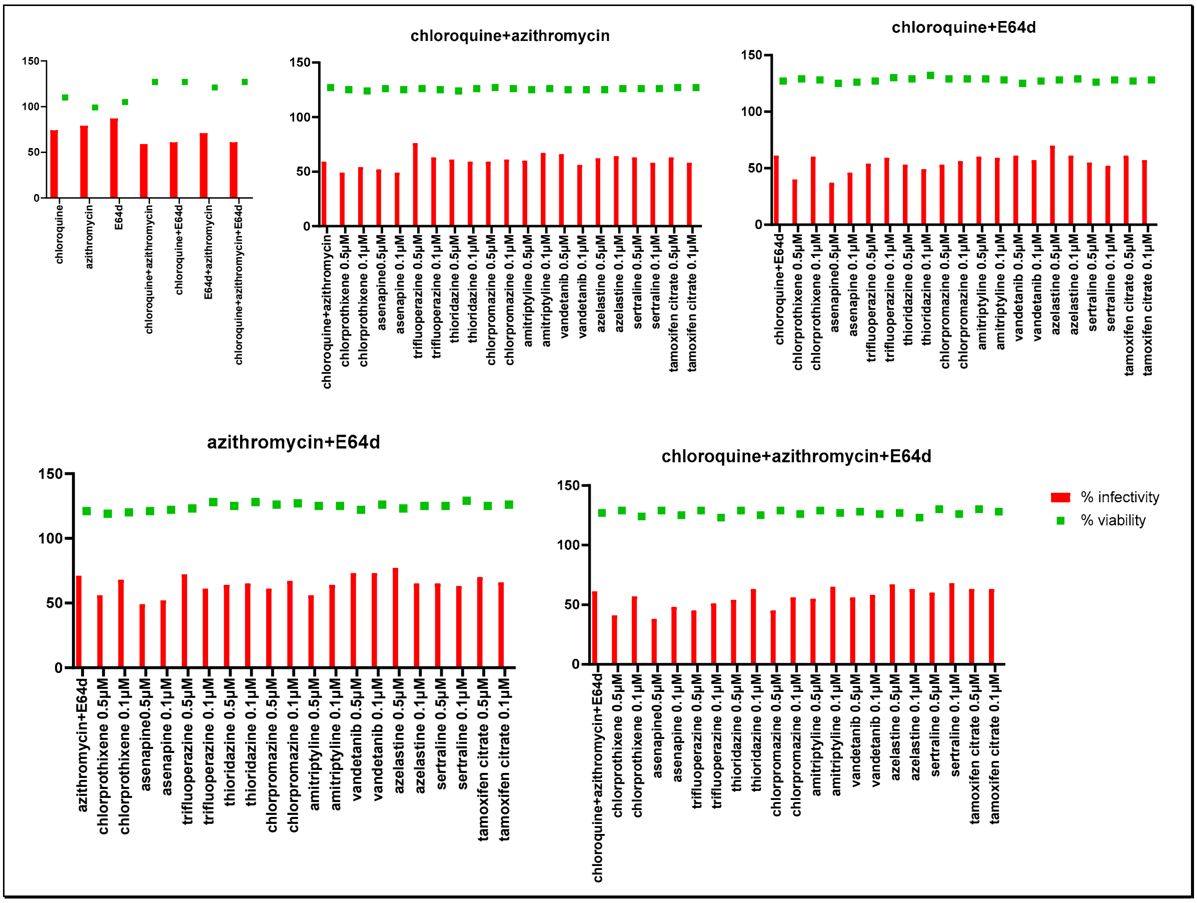

Figure S7. **Drug hits show additive inhibitory effect on SARS-CoV-2 infectivity.** Mouse leukaemia virus pseudotyped with spike protein (S) from severe acute respiratory syndrome coronavirus-2 was used to infect 293T-ACE2 cells in 96-well plate for 48h in the presence of the drug combination in the specified doses, as indicated, with 1h pre-treatment. Infectivity was measured as luciferase activity and expressed as % infectivity to infected, own solvent control (dimethylsulphoxide or water). Viability was measured by XTT assays in un-infected

cells and expressed as % viability to un-infected solvent control (dimethylsulphoxide or water). Data are from one repeat.

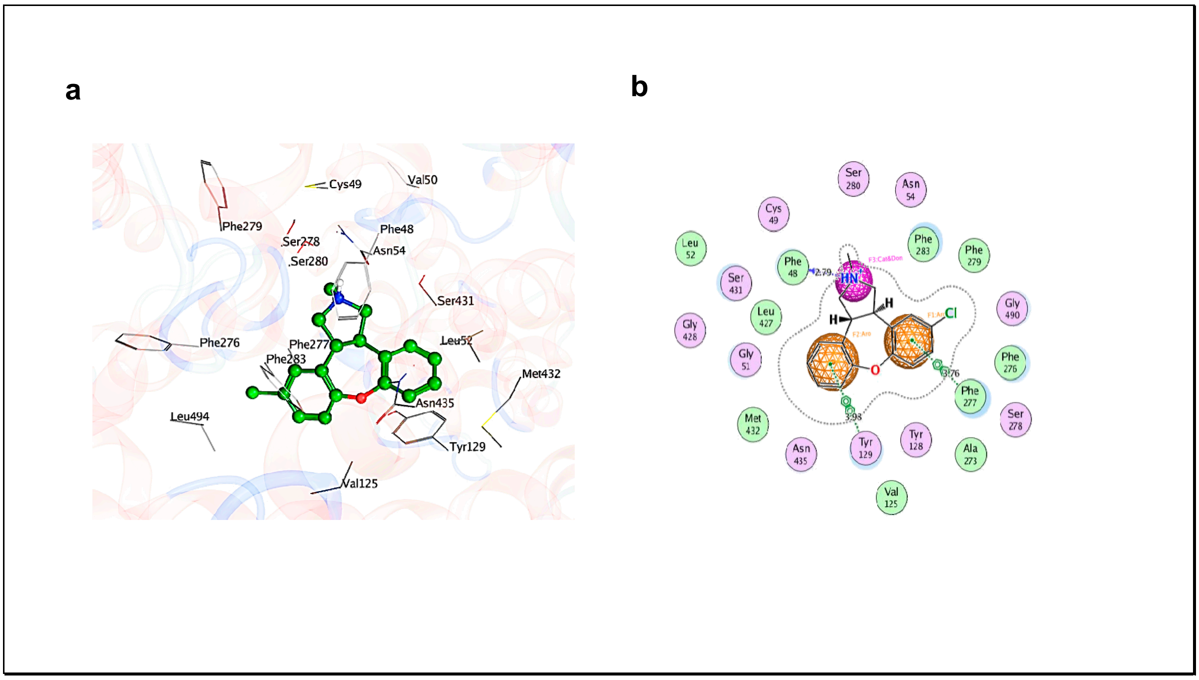

Figure S8. diagrammatic representation of ligand- protein interaction and pharmacophore mapping. a) Ligand protein profile of the highest ranked conformation of asenapine within the SLC6a19 binding cavity. b) Two dimensional representation of ligand protein interaction of asenapine overlaid with the pharmacophore model.

References

1. Renner, T. M., Tang, V. A., Burger, D., and Langlois, M. A. (2020) Intact Viral Particle Counts Measured by Flow Virometry Provide Insight into the Infectivity and Genome Packaging Efficiency of Moloney Murine Leukemia Virus. *J Virol* **94**
